# TORC1 signaling modulates Cdk8-dependent *GAL* gene expression in *Saccharomyces cerevisiae*

**DOI:** 10.1093/genetics/iyab168

**Published:** 2021-10-05

**Authors:** Riley Horvath, Nicole Hawe, Cindy Lam, Konstantin Mestnikov, Mariam Eji-Lasisi, John Rohde, Ivan Sadowski

**Affiliations:** 1 Department of Biochemistry and Molecular Biology, Molecular Epigenetics Group, LSI, University of British Columbia, Vancouver, BC V6T 1Z3, Canada; 2 Department of Microbiology and Immunology, Dalhousie University, Halifax, NS B3H 4R2, Canada

**Keywords:** yeast, *GAL* genes, Gal4, Cdk8, Tor, PP2A, Cdc55, phosphorylation, transcription

## Abstract

Cdk8 of the RNA polymerase II mediator kinase complex regulates gene expression by phosphorylating sequence-specific transcription factors. This function is conserved amongst eukaryotes, but the signals and mechanisms regulating Cdk8 activity and phosphorylation of its substrates are unknown. Full induction of the *GAL* genes in yeast requires phosphorylation of the transcriptional activator Gal4 by Cdk8. We used a screen to identify regulators of the Cdk8-dependent phosphorylation on Gal4, from which we identified multiple mutants with defects in TORC1 signaling. One mutant, designated gal four throttle 1 (*gft1*) was identified as a recessive allele of *hom3*, encoding aspartokinase, and mutations in *hom3* caused effects typical of inhibition of TORC1, including rapamycin sensitivity and enhanced nuclear localization of the TORC1-responsive transcription factor Gat1. Mutations in *hom3* also inhibit phosphorylation of Gal4 *in vivo* at the Cdk8-dependent site on Gal4, as did mutations of *tor1*, but these mutations did not affect activity of Cdk8 assayed *in vitro*. Disruption of *cdc55*, encoding a regulatory subunit of the TORC1-regulated protein phosphatase PP2A, suppressed the effect of *hom3 and tor1* mutations on *GAL* expression, and also restored phosphorylation of Gal4 at the Cdk8-dependent site *in vivo*. These observations demonstrate that TORC1 signaling regulates *GAL* induction through the activity of PP2A/Cdc55 and suggest that Cdk8-dependent phosphorylation of Gal4 is opposed by PP2A/Cdc55 dephosphorylation. These results provide insight into how induction of transcription by a specific inducer can be modulated by global nutritional signals through regulation of Cdk8-dependent phosphorylation.

## Teaser

Horvath, Hawe *et al*. examine regulators of Cdk8-dependent Gal4 activity in a screen for defects in the LTA response of *gal3* yeast. They identify TORC1 components *tor1* and *tco89* and the downstream PP2A/Cdc55 phosphatase as regulators of Gal4. Additionally, genetic analysis indicates that mutations in *hom3*, which encodes aspartate kinase, produce defects in TOR signaling. These results demonstrate that regulation of Gal4 by phosphorylation is modulated by the TOR nutritional signaling pathway through the activity of PP2A/Cdc55.

## Introduction

Cdk8 is a protein kinase of the eukaryotic RNA polymerase II mediator complex that modulates gene expression in response to sequence-specific transcription factors. Phosphorylation of transcriptional activators by Cdk8 produces positive or negative effects on gene expression, depending on the functional effect of the modification (Nemet *et al.* 2014). The role of Cdk8 for both repression and activation of gene expression is conserved in humans, but is best understood in yeast, where mutants of *cdk8* were identified in numerous screens for alterations in gene regulation, resulting in a proportion of aliases that reflects its diverse effect on gene regulation. These include suppressors of *snf1* (*ssn3)* (Carlson *et al.* 1984), suppressors of RNA polB (*srb10)* (Hengartner *et al.* 1998; Liao *et al*. 1995), unscheduled meiosis (*ume5)* (Surosky *et al.* 1994), glucose inhibition of gluconeogenic genes (*gig2)* (Balciunas and Ronne 1995), negative regulation of (*HO*) URS2 (*nut7)* (Tabtiang and Herskowitz 1998), suppressors of *swi6 (ssx7)* (Li *et al*., 2005), URS_G_-mediated repression *(urr1)* (Flick and Johnston, 1992), and regulation of *YGP1* expression *(rye5)* (Kuchin and Carlson, 1998). Cdk8 activity is dependent upon additional proteins of the mediator kinase module, including cyclin C/Srb11, Srb8/Med12, and Srb9/Med13 (Klatt *et al.* 2020). Additionally, observations indicate that Cdk8 activity in yeast is regulated by nutrient availability. Cdk8 is degraded in nitrogen starved cells (Nelson *et al.* 2003), and its abundance is reduced as nutrients become depleted (Hengartner *et al.* 1998). The associated cyclin C/Srb11 is also degraded in response to oxidative stress and carbon limitation (Cohen *et al.* 2003). Remarkably, despite its role in regulating responses to multiple physiological signals in yeast, a function that is likely conserved in humans (Dannappel *et al*., 2018), signaling mechanisms that regulate Cdk8 and phosphorylation of its substrates have not been identified.

Several Cdk8 transcription factor substrates in yeast, including Ste12 and Phd1 (Raithatha *et al.* 2012), regulate filamentous growth (FG) in response to nutrient limitation, where cells differentiate into elongated filamentous cells to promote nutrient foraging (Cullen and Sprague 2012). Nitrogen limitation inhibits Cdk8 activity (Nelson *et al.* 2003), which allows accumulation of these factors to cause induction of genes that promote FG. In contrast, other transcriptional activators, notably Gal4, are positively regulated by Cdk8 phosphorylation (Hirst *et al.* 1999), where phosphorylation is required for full induction of the *GAL* genes in response to galactose (Rohde *et al.* 2000). Additional yeast transcriptional activators that are positively regulated by Cdk8 include Sip4 (Vincent *et al.* 2001) and Skn7 (Aristizabal *et al.* 2019). For each of these regulatory effects, activity of Cdk8 is associated with a favorable growth environment, and Cdk8 activity, and phosphorylation of its substrates are inhibited under conditions of nutrient or physiological stress (Rohde *et al.* 2000; Nelson *et al.* 2003), although as mentioned, signaling mechanisms that regulate Cdk8 activity have not been identified.

Yeast respond to their nutritional environment through multiple signaling mechanisms that include the RAS-cAMP-PKA, SNF1/AMPK, and target of rapamycin (TOR) pathways, which are interconnected by cross-talk, and are conserved amongst eukaryotes (Smets *et al.* 2010). TOR signaling regulates cell growth by promoting macromolecular synthesis in response to nutrients, particularly amino acids, while inhibiting catabolic processes and autophagy (Kim and Guan 2019). TOR-complex 1 (TORC1), comprised of the partially redundant Tor1 or Tor2 protein kinases, and the regulatory subunits Lst8, Kog1, and Tco89, responds to the abundance and quality of nutrients, primarily nitrogen (Loewith and Hall 2011), and is inhibited by the fungal antibiotic rapamycin in a complex with the prolyl isomerase FKBP12/Fpr1, to produce an effect that mimics nutrient starvation in both yeast and human cells (González and Hall 2017). TORC1 activity stimulates protein translation, ribosome biogenesis, cell cycle progression, and regulates nutrient uptake and amino acid metabolism (Loewith and Hall 2011). These responses are mediated by multiple downstream targets, including Sch9 (Urban *et al.* 2007), the yeast homologue of ribosomal protein S6 kinase, and Tap42, which regulates the activity of two protein phosphatase complexes, the type 2A phosphatase Pph21/Pph22/Pph3, and 2A-related phosphatase Sit4 (Düvel *et al.* 2003; Conrad *et al.* 2014). A significant proportion of transcriptional responses to nutrients regulated by TORC1 involves dephosphorylation of transcription factors by these phosphatases. For example, inhibition of TORC1 by nitrogen limitation causes activation of PP2A and Sit4 which dephosphorylate the transcription factors Gat1 and Gln3, allowing translocation to the nucleus for activation of genes that are normally repressed under ideal growth conditions, an effect known as nitrogen catabolite repression (Georis *et al.* 2008). Most studies of transcriptional responses involving TOR have focused on nitrogen availability, but TORC1 is also inhibited by additional stress conditions, including glucose limitation (González and Hall 2017). Acute glucose depletion causes rapid reorganization of TORC1 into large vacuole-associated multimerized complexes (Hughes Hallett *et al.* 2015), in a process dependent upon the Gtr1/Gtr2 GTPases, and which is associated with a corresponding loss of Sch9 phosphorylation (Prouteau *et al.* 2017). However, overall the mechanism(s) by which glucose and carbon signaling modifies activity of transcription factors regulated downstream of TORC1 have not been characterized.

The genes required for utilization of galactose (*GAL*) in yeast have served as an important model for understanding mechanisms of transcriptional regulation in eukaryotes (Das Adhikari *et al.* 2014). The *GAL* genes are activated by Gal4, which in the absence of galactose is inhibited by interaction with Gal80 (Li *et al.* 2010). Galactose induces *GAL* expression as a ligand of Gal3 protein, which relieves the inhibitory effect of Gal80 on Gal4 (Kar *et al.* 2017). Yeast with *gal3* mutations produce a distinctive phenotype where *GAL* expression is induced only after several days exposure to galactose instead of minutes to hours in WT yeast, an effect termed long-term adaptation (LTA) to galactose (Bhat *et al.* 1990). Characterization of the *gal3* LTA phenotype revealed that full *GAL* induction requires phosphorylation of Gal4 by Cdk8 at S699, and consequently that *GAL* expression is modulated by signals that control Cdk8 (Rohde *et al.* 2000), although as mentioned, signals controlling Cdk8 or phosphorylation of its substrates have not been identified.

To identify mechanisms regulating Cdk8, we conducted a genetic screen that exploited the dependence of Cdk8 for growth of *gal3* yeast on galactose. From this effort, we identified mutants that prevent *GAL* expression only in combination with a *gal3* null allele, designated the gal four throttle mutants (*gft*). One class of this mutant collection revealed that phosphorylation of Gal4 by Cdk8 is opposed by PP2A/Cdc55 phosphatase downstream of TORC1. Here, we demonstrate that *GAL* induction is modulated by a mechanism involving TORC1 signaling and a balance of phosphorylation of the regulatory Cdk8-dependent site on Gal4.

## Materials and methods

### Yeast strains, plasmids, yeast techniques, and immunoblotting

All strains were derived from the W303-1A background and are detailed in Table 1, and DNA plasmids described in Table 2. Gene disruptions and reporter gene integrations were produced by homologous recombination using standard PCR-based methods and confirmed by PCR analysis of chromosomal DNA (Longtine *et al.* 1998). Additional yeast genetic manipulations were performed as described (Dunham *et al.* 2015). Protein samples for immunoblotting were prepared by extraction with LiAc and NaOH (Zhang *et al.* 2011). Antibodies against yeast aspartate kinase (Hom3) were produced in rabbits against a GST-Hom3 fusion protein expressed and purified from *E. coli*. Antibodies against the Gal4 DNA-binding domain were produced in rabbits against 6-His-Gal4 (1–147) purified from *E. coli*. The Hom3 D234A mutation in plasmid pNH01 was created by site directed mutagenesis using oligos NH01 (5′-GGCTATACCGcaTTATGTGCCG-3′) and NH02 (5′-ACGACCAACACCATTCAG-3′) with the NEB Q5 SDM system. Plates with nonfermentable carbon sources contained 5% glycerol, 2% lactic acid, and 2% ethanol. Ethidium bromide (EB) galactose plates (EB gal) contained yeast extract-peptone supplemented with 2% galactose and 20 mg of EB per liter. YPD/AOA and EB gal/AOA plates contained 10 nM aminooxyacetic acid (AOA) (Sigma-Aldrich). Rapamycin was added to YPD plates at 5 ng/ml.

**Table 1:** Yeast strains**^*a*^**

Strain	Genotype	Ref.
W303-1a	MATa *ade2 his3 LEU2 trp1 ura3 can1*	Ralser *et al.* (2012)
YJR5	*MATa ade2 his3 leu2 trp1 ura3 can1 URA3*::*pGAL1*-lacZ	Rohde *et al.* (2000)
YJR7::131	*MAT*a *ade2 his3 leu2 trp1 ura3 can1 gal3::LEU2 URA3::pGAL1-LacZ*	Rohde *et al.* (2000)
YJR40	MATa *trp1 ura3 leu2 his3 gal4 gal3:*:*LEU2 pGAL1*-LacZ::*URA3*	This study
YJR52	*MAT*α *ade2 his3 leu2 trp1 ura3 can1 gal3::LEU2 HIS3*	This study
ISY54	MATa *ade2 his3 LEU2 trp1 ura3 can1 gal3::LEU2*	This study
ISY128	MATa *ade2 his3 LEU2 trp1 ura3 can1 gal3::LEU2, hom3::HIS3*	This study
ISY135	MATa *can1 ade2 his3 leu2 trp1 gal3*::*LEU2 gft1-1 URA3*::p*GAL1*-LacZ	This study
ISY136	MATa *ade2 his3 LEU2 trp1 ura3 can1 fpr1::URA3*	This study
ISY137	MATa *ade2 his3 LEU2 trp1 ura3 can1 gal3::LEU2 hom3::HIS3 fpr1::URA3*	This study
ISY138	MATa *ade2 his3 LEU2 trp1 ura3 can1 gal3::LEU2 fpr1::URA3*	This study
ISY158	MATa *HIS3 can1 ade2 leu2 trp1 ura3 gal3*::*LEU2 gft2-1*	This study
ISY161	MATa *HIS3 can1 ade2 leu2 trp1 ura3 gal3*::*LEU2 gft6-1*	This study
ISY162	MATa *HIS3 can1 ade2 leu2 trp1 ura3 gal3*::*LEU2 gft7-1*	This study
ISY170	MATa *HIS3 can1 ade2 leu2 trp1 ura3 gal3*::*LEU2 gft14-1*	This study
ISY187	MATα *HIS3 can1 ade2 leu2 trp1 ura3 gal3*::*LEU2 gft2-1*	This study
ISY191	MATα *HIS3 can1 ade2 leu2 trp1 ura3 gal3*::*LEU2 gft6-1*	This study
ISY192	MATα *HIS3 can1 ade2 leu2 trp1 ura3 gal3*::*LEU2 gft7-1*	This study
ISY200	MATα *HIS3 can1 ade2 leu2 trp1 ura3 gal3*::*LEU2 gft14-1*	This study
ISY277	MATa *ade2 his3 LEU2 trp1 ura3 can1 gal3::LEU2 hom2::HIS3*	This study
ISY279	MATa *ade2 his3 LEU2 trp1 ura3 can1 hom3::HIS5*	This study
ISY281	MATa *ade2 his3 LEU2 trp1 ura3 can1 gal3::LEU2 sit4::kanMX6*	This study
ISY282	MATa *ade2 his3 LEU2 trp1 ura3 can1 gal3::LEU2 sit4::kanMX6 hom3::HIS3*	This study
ISY311	MATa *ade2 his3 LEU2 trp1 ura3 can1 hom2::HIS3*	This study
ISY396	*MAT*a*/*α *ade2/ade2, can1/can1, leu2/leu2, trp1/trp1, ura3/ura3, his3/HIS3 gal3::LEU2/gal3::LEU2, tor1::kanMX6/TOR1, URA3::GAL1-LacZ*	This study
ISY397	MATa *ade2 his3 LEU2 trp1 ura3 can1 Nab2-*mCherry*::TRP1*	This study
ISY398	MATa *ade2 his3 LEU2 trp1 ura3 can1 gal3::LEU2 Nab2-*mCherry*::TRP1*	This study
ISY399	MATa *ade2 his3 LEU2 trp1 ura3 can1 hom3::HIS5 Nab2-*mCherry*::TRP1*	This study
ISY400	MATa *ade2 his3 LEU2 trp1 ura3 can1 gal3::LEU2, hom3::HIS3 Nab2-*mCherry*::TRP1*	This study
ISY401	MATa *ade2 his3 LEU2 trp1 ura3 can1 gal3::LEU2 gft1-1 Nab2-*mCherry*::TRP1*	This study
ILY002	MATa *ade2 his3 leu2 trp1 ura3 can1 hom3::HIS3 URA3::pGAL1-LacZ*	This study
YNH003	MATa *ade2 his3 LEU2 trp1 ura3 can1 srb10::HIS5*	This study
YNH008	MATa *ade2 his3 LEU2 trp1 ura3 can1 tor1::kanMX6*	This study
YNH009	MATa *can1 ade2 his3 LEU2 trp1 ura3 gal3::LEU2 tor1::kanMX6*	This study
YNH010	MATa *can1 ade2 his3 LEU2 trp1 ura3 cdc55::kanMX6*	This study
YNH011	MATa *can1 ade2 his3 LEU2 trp1 ura3 gal3::LEU2 hom3::HIS3 cdc55::kanMX6*	This study
YNH014	MATa *can1 ade2 his3 LEU2 trp1 ura3 hom6::kanMX6*	This study
YNH015	MATa *can1 ade2 his3 LEU2 trp1 ura3 gal3::LEU2 hom6::kanMX6*	This study
YNH016	MATa *can1 ade2 his3 LEU2 trp1 ura3 tco89::kanMX6*	This study
YNH017	MATa *can1 ade2 his3 LEU2 trp1 ura3 gal3::LEU2 tco89::kanMX6*	This study
YNH019	MATa *ade2 can1 his3 LEU2 trp1 ura3 gal3::LEU2 tor1::kanMX6 cdc55::His3MX6*	This study
YNH024	MATa *ade2 his3 ura3 leu2 trp1 can1 srb10::HIS3 gal3::LEU2*	This study
YNH025	MATa *ade2 his3 ura3 leu2 trp1 can1 gal3::LEU2 cdc55::KanMX6*	This study
YNH026	MATa *ade2 his3 ura3 leu2 trp1 can1 gal3::LEU2 srb10::KanMX6 cdc55::HIS3*	This study
YNH028	MATa *ade2 his3 ura3 leu2 trp1 can1 gal3::LEU2 tco89::kanMX6 cdc55::HIS3*	This study
YNH029	MATa *ade2 his3 leu2 trp1 ura3 can1 ade8::TRP1: pGAL1-*GFP	This study
YNH030	MATa *can1 ade2 his3 LEU2 trp1 ura3 gal3::LEU2 ade8::TRP1: pGAL1-*GFP	This study
YNH031	MATa *can1 ade2 his3 leu2 trp1 ura3 hom3::HIS5 ade8::TRP1: pGAL1-*GFP	This study
YNH032	MATa *can1 ade2 his3 leu2 trp1 ura3 gal3::LEU2 hom3::HIS5 ade8::TRP1: pGAL1-*GFP	This study
YNH033	MATa *can1 ade2 his3 leu2 trp1 ura3 tor1::kanMX6 ade8::TRP1: pGAL1-*GFP	This study
YNH034	MATa *can1 ade2 his3 leu2 trp1 ura3 gal3::LEU2 tor1::kanMX6 ade8::TRP1: pGAL1-*GFP	This study
YNH035	MATa *can1 ade2 his3 leu2 trp1 ura3 tco89::kanMX6 ade8::TRP1: pGAL1-*GFP	This study
YNH036	MATa *can1 ade2 his3 leu2 trp1 ura3 gal3::LEU2 tco89::*kanMX6 *ade8::TRP1: pGAL1-*GFP	This study
YNH037	MATa *trp ura3 leu2 his4 gal4 gal3*::LEU2 p*GAL1*-LacZ::*URA3 cdc55*::*HIS3*	This study
YKM001	*MATa ade2 his3 leu2 trp1 ura3 can1 gal80*::hphMX6	This study
YKM002	*MATa can1 ade2 his3 leu2 trp1 ura3 cdc55*::kanMX6 *gal80*::hphMX6	This study
YKM003	*MATa ade2 can1 his2 leu2 trp1 ura3 tor1*::kanMX6 *gal80*::hphMX6	This study
YKM004	*MATa can1 ade2 his3 leu2 trp1 gal3*::*LEU2 gft1-1 URA3*::*GAL1*-LacZ *gal80*::hphMX6	This study
YKM005	*MATa ade2 can1 his2 leu2 trp1 ura3 can1 hom3*::HIS5 *gal80*::hphMX6	This study
YKM010	*MATa can1 ade2 his3 leu2 trp1 ura3 gal3::LEU2 gal80::hphMX6*	This study
YKM013	*MATa ade2 can1 his2 leu2 trp1 ura3 can1 hom3*::HIS5 *gal80*::hphMX6 *cdc55::HIS5*	This study
YKM014	*MATa ade2 can1 his2 leu2 trp1 ura3 tor1*::kanMX6 *gal80*::hphMX6 *cdc55::HIS5*	This study
YKM016	MATa *ade2 his3 LEU2 trp1 ura3 can1 gal3::LEU2 gft1-1*	This study
YKM017	*MAT*a*/*α *ade2/ade2, can1/can1, leu2/leu2, trp1/trp1, ura3/ura3, his3/HIS3 gal3::LEU2/gal3::LEU2, tor1::kanMX6/TOR1, gft6-1/GFT6*	This study
YKM018	*MAT*a*/*α *ade2/ade2, can1/can1, leu2/leu2, trp1/trp1, ura3/ura3, his3/HIS3 gal3::LEU2/gal3::LEU2, tor1::kanMX6/TOR1, gft2-1/GFT2*	This study
YKM019	*MAT*a*/*α *ade2/ade2, can1/can1, leu2/leu2, trp1/trp1, ura3/ura3, his3/HIS3 gal3::LEU2/gal3::LEU2, tor1::kanMX6/TOR1, gft14-1/GFT14*	This study

a All strains are derived from W303-1A.

**Table 2:** Plasmid DNAs

Plasmid	Description	Ref.
pRS314	*TRP1*; ARS-CEN	Sikorski and Hieter (1989)
pIS556	*TRP1*; ARS-CEN; TEF1-Hom3-3X FLAG-6	This study
pIS574	*TRP1*; ARS-CEN; TEF1-Hom3	This study
pNH01	*TRP1*; ARS-CEN; TEF1-Hom3 (D234A)	This study
pIS297	*TRP1*; ARS-CEN; *HOM3* (clone 1)	This study
pIS298	*TRP1*; ARS-CEN; *HOM3* (clone 2)	This study
pIS299	*TRP1*; ARS-CEN; *HOM3* (clone 3)	This study
pIS484	*URA3*; ARS-CEN; TEF1-Srb10/Cdk8-3X-FLAG-6his	This study
pIS686	pBSKSII—*gft1-1* (ORF ±1 kb)	This study
pIS698	pGEM11Z—*gft7-1* (ORF ±1 kb)	This study
pIS528	*URA3*; ARS-CEN; TEF1-Srb10/Cdk8 (D290A)-3X-FLAG-6his	This study
YCpG4trp	*TRP1*; ARS-CEN; *GAL4*	Sadowski *et al.* (1996)
pRD038	*TRP1*; ARS-CEN; *GAL4 (S699A)*	Sadowski *et al.* (1996)
pKW10△683	*TRP1*; ARS-CEN; *GAL4*(△148–682)	Sadowski *et al.* (1996)
pMH683A699	*TRP1*; ARS-CEN; *GAL4*(△148–682)S699A	Sadowski *et al.* (1996)
pRD021	*TRP1*; ARS-CEN; *GAL4*(△148–682)S837A	Sadowski *et al.* (1996)
pRS416-GAT-GFP	*URA3*; ARS-CEN; Gat1-GFP	Tate *et al.* (2015)
pJP015	*URA3*; ARS-CEN; TEF1-Srb10/Cdk8-GFP	This study
pJR015	*HIS3;* ARS-CEN*; GAL3*	This study
pZOM82	*TRP1: Nab2-*mCherry	Zhu *et al.* (2019)

### Mutagenesis of yeast, complementation analysis, and characterization of *gft* alleles

The screen for defects in Cdk8-dependent *GAL* induction, gal four throttle (*gft*), was performed by UV mutagenesis of *gal3* W303 strain YJR7::131; colonies of mutagenized cells, grown on minimal medium containing glycerol, ethanol, and lactic acid as the sole source of carbon, were replica plated onto EB gal plates. Mutants incapable of growth on EB gal were transformed with a plasmid expressing WT *GAL3* and re-examined for growth on EB gal; mutants incapable of growth when *GAL3*^+^ likely represented *gal* mutants and were discarded. Diploids were produced with the remaining mutant strains by crossing with an *MAT*α *gal3* strain (YJR52); all of the mutants analyzed produced a recessive *gft* phenotype in that the resulting diploids grew on EB gal as well as did the parental WT *gal3* strain (Supplementary Figure S9). Mutants that produced a 2:2 ratio of *gal3* WT: *gft* phenotype on EB gal in tetrad analysis were characterized further. The mutant collection was organized into 17 complementation groups, and each of the *gft* mutants described here were back-crossed to WT *gal3* W303 a minimum of six times. A complete description of the additional *gft* mutants and their respective phenotypes will be described elsewhere.

Identity of the *gft1-1* mutation was determined using complementation by transformation with a library prepared from WT DNA in pRS314 (*TRP1*, *ARS-CEN*). Transformants were selected on SC-trp plates and the resulting colonies replica plated onto EB gal. Colonies that grew on EB gal were re-streaked on SC-trp and re-examined for growth on EB gal. Library plasmids were recovered from EB gal^+^ clones by transformation into DH5α *E. coli*, and analyzed by sequencing with the T7 (5′-TAATACGACTCACTATAGGG-3′) and T3 (5′-ATTAACCCTCACTAAAG-3′) promoter sequencing primers. Of the five clones sequenced, four contained DNA fragments spanning a region containing *HOM3* on chromosome V (Supplementary Figure S1), and one bore a DNA segment spanning *GAL3* from chromosome IV.

Clones of the *gft1-1* allele were isolated by amplification from genomic DNA with oligos IS2897 (5′-ccgGCGGCCGCTAAGAATACTGCTGGATAATTATTTTAT-3′) and IS2898 (5′-cggCTCGAGACCCTGACATTACATTTAGGGAA-3′), digestion with *Xho*I/*Not*I and cloning into pBSKSII. Similarly, *gft7-1* clones were produced by amplification with oligos IS2899 (5′-ccgGCGGCCGCTGGCACGATGATTAAGG-3′) and IS2900 (5′-ccgGGATCCCGTTTTACCTCGAAAGCATTAGCTA-3′), digestion with *Bam*HI/*Not*I and cloning into pGEM11Z. Multiple clones from each ligation and cloning were sequenced with nested sets of forward and reverse primers specific for the *HOM3 and TCO89* ORF and 1 kb flanking DNA, respectively.

### Flow cytometry, reporter gene quantitation, and fluorescence microscopy

Strains with *GAL1*-GFP reporters were grown at 30°C in SC media containing glycerol, lactic acid, and ethanol as the sole carbon source, to an OD *A*_600_ ∼ 1.0 and then induced with 2% galactose. Samples were taken at the indicated times and diluted in PBS to 400,000 cells/ml and analyzed for GFP expression and side scatter, indicating cellular granularity produced by internal cellular complexity and physiological state, using a Guava Easycyte Flow Cytometer (MilliporeSigma). Mean fluorescence intensity (MFI) was calculated using FlowJo software. Expression of the *GAL1*-LacZ reporter was determined by assay of β-galactosidase activity from permeabilized cells as previously described (Dunham *et al.* 2015).

To monitor effects on Gat1 subcellular localization, WT, *gal3*, *hom3, gal3 hom3*, and *gal3 gft1-1* strains expressing Nab2-mCherry (Zhu *et al.* 2019) were transformed with plasmid pRS416GAT1 (*URA3*, *ARS-CEN*), expressing *GAT1*-GFP or pJP01, expressing *CDK8*-GFP. Cells were grown in SC medium with glycerol, ethanol, and lactic acid or 2% raffinose as carbon source to an OD *A*_600_ ∼ 1.0 and then induced with 2% galactose for 2 h. Cells were recovered by centrifugation, washed in PBS, suspended in 1/4 original volume PBS, and examined using a Leica laser scanning confocal microscope; GFP and mCherry images were captured in separate channels for analysis using Image J. Cells expressing nuclear Gat1-GFP was quantitated using the Image J cell counter plugin, and represents the proportion of cells that have concentrated GFP expression co-localized with Nab2-mCherry (Figure 5A). Results were determined from a minimum of 3 images with at least 120 cells, captured from 2 independent cultures.

### Protein kinase assays

To measure Cdk8 kinase activity *in vitro*, yeast with the indicated genotype expressing Srb10/Cdk8-3XFLAG, or kinase inactive D290A Cdk8, were grown in SD-ura to an OD *A*_600_ ∼ 0.8 and recovered by centrifugation. Cells were washed twice in kinase lysis buffer (KLB) [50 mM Tris (pH 7.5), 5 mM ETDA, 200 mM NaCl, 0.1% NP-40, and protease inhibitors], and resuspended in 1 ml KLB/100 ml culture. Cells were lysed by vortexing with glass beads, and the lysates clarified by centrifugation at 13,000×*g* for 10 min at 4°C. FLAG-tagged Srb10/Cdk8 was recovered by immunoprecipitation with anti-FLAG-conjugated M2 agarose beads (Sigma). Samples were washed two times in KLB, twice in kinase assay buffer (KAB) [10 mM MgCl2, 50 mM Tris (pH 7.5), 1 mM DTT and protease inhibitors], and the beads suspended in 50 μl KAB. Reactions contained 5 μl of the bead suspension and 1 μg substrate protein in 10 μl KAB. Two picomoles of [γ-32P] ATP were added, and the reactions incubated at 30°C for 20 min. Reactions were stopped by addition of 10 μl 2× SDS-PAGE sample buffer, boiled for 2 min, and resolved on 10% SDS-PAGE. The gels were stained with Coomassie blue (Supplementary Figure S12), dried, and exposed to Kodak Biomax film. GST, and GST-CTD substrate protein were expressed and purified from *E. coli* (Aristizabal *et al.* 2019), and WT Gal4 from insect cells using baculovirus (Hirst *et al.* 1999).

## Results

### A minor subset of *gal3* yeast induce full *GAL* expression in response to galactose

**Figure 1:**
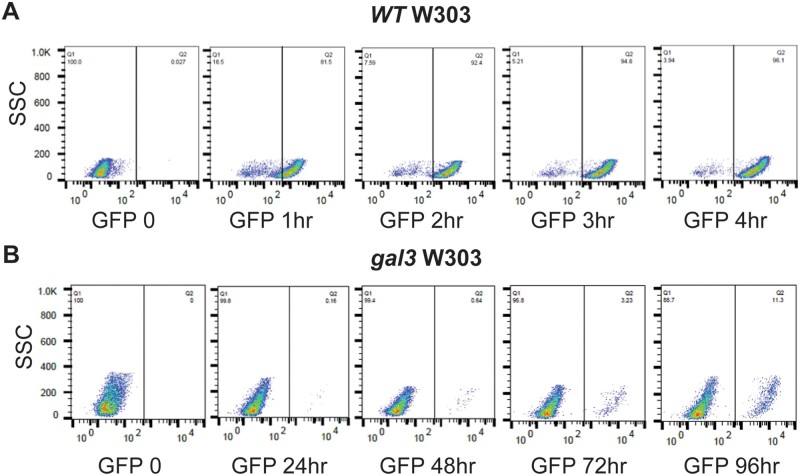
Yeast defective for *gal3* produce delayed induction of a *GAL1-GFP* reporter gene. Wild-type W303-1A yeast (yNH029) (A) or *gal3* (yNH30) (B) bearing a *GAL1*-GFP reporter gene were grown in minimal media containing glycerol, lactic acid and ethanol (GFP 0), and induced by addition of galactose to 2% for the indicated time (hours) when samples were taken for analysis by flow cytometry. GFP fluorescence is indicated on the *X*-axis and side scatter, indicative of cell granularity, on the *Y*-axis.

**Figure 2:**
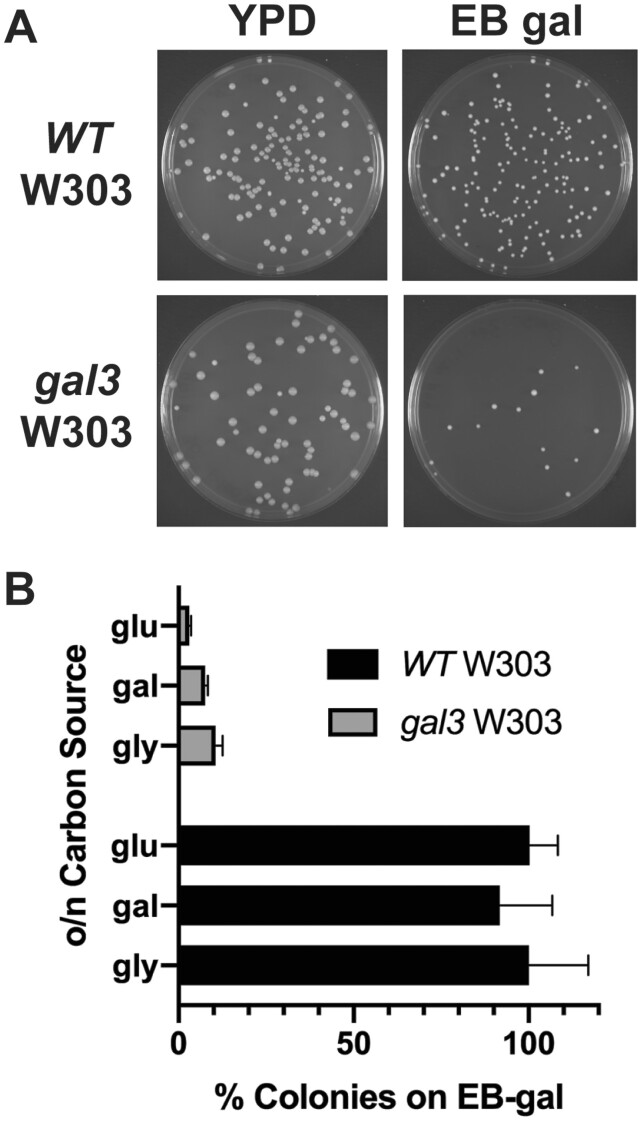
Yeast defective for *gal3* produce rare but robust colonies on EB gal plates, typical of the LTA phenotype. (A) WT and *gal3* (ISY54) yeast were grown overnight in YPD, diluted to equivalent OD *A*_600_, plated on YPD (left) or EB gal plates (right), and incubated at 30°C for 5 days. (B) WT and *gal3* (ISY54) yeast were grown overnight in YP media containing glucose (glu), galactose (gal), or glycerol/ethanol and lactic acid (gly) as carbon source. Cells were diluted to an equivalent OD *A*_600_, plated on YPD or EB gal plates and incubated at 30°C for 5 days. Results indicate the percentage of colonies formed on EB gal relative to YPD, and represent an average from three independent cultures.

Yeast defective for *gal3*, encoding the galactose receptor/inducer protein, produce a distinctive LTA phenotype where induction of *GAL* expression occurs days post exposure to galactose rather than minutes to hours as in wild-type yeast (Bhat *et al.* 1990). Hence, with wild-type yeast (W303) bearing a GFP reporter expressed from the *GAL1* promoter we find that ∼80% of cells induce significant GFP expression within an hour of galactose addition, and greater than 95% become fully induced within 4 h (Figure 1A). In contrast, consistent with previous observations (Kar *et al.* 2014), only a minor subset of *gal3* yeast induce GFP expression even at 24 h, and after 96 h only ∼10% of the cells induce significant expression (Figure 1B). Importantly, however, individual *gal3* cells eventually induce GFP expression comparable to levels similar to wild-type. Robust induction of *GAL* expression in a minor proportion of *gal3* yeast is also observed by growth on plates containing EB, with galactose as the sole carbon source (EB gal). EB inhibits mitochondrial function and forces utilization of sugars as carbon source by fermentation (Kar *et al.* 2014); consequently, growth on EB gal is a stringent measurement of *GAL* gene induction. Consistent with results using the GFP reporter, we find that *gal3* W303 yeast form colonies on EB gal plates of similar size as wild-type, but at significantly lower frequency (Figure 2A). Typically, 10% or less *gal3* W303 cells produce colonies on EB gal, depending on the carbon source on which plated cells were initially grown, in contrast to WT cells which form colonies on EB gal at ∼100% efficiency (Figure 2B).

Importantly, we have previously shown that disruption of *cdk8* or mutation of the Cdk8-dependent phosphorylation site on Gal4 at S699 completely prevents induction of *GAL* gene expression by galactose, and growth on EB gal plates (Rohde *et al.* 2000).

### *Hom3*/*gft1* is necessary for LTA/delayed induction of *gal3* yeast

**Figure 3:**
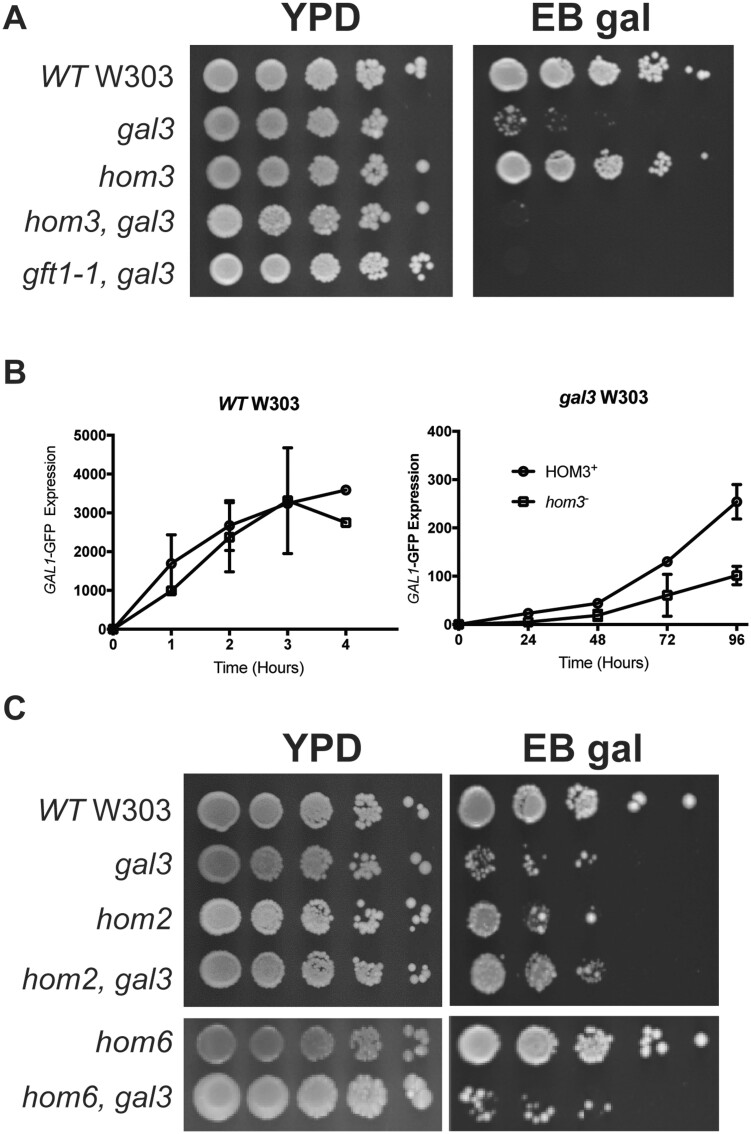
Aspartate kinase/Hom3 is required LTA. (A) Strains with the indicated genotype were grown overnight in YPD, diluted to an OD *A*_600_ of 1.0, spotted onto YPD or EB gal plates in 10-fold serial dilutions, and grown at 30°C for 5 days. (B) WT (left) or *gal3* (right) yeast bearing a *GAL1*-GFP reporter gene and expressing WT *HOM3* (yNH029, yNH030) (●) or a *hom3* disruption (yNH031, yNH032) (■) were induced with 2% galactose for the indicated time (hours) and analyzed by flow cytometry. Results are presented as MFI of GFP expression and represent an average from three independent cultures. (C) Strains with the indicated genotype were diluted, spotted onto YPD or EB gal plates in 10-fold serial dilution and grown at 30°C for 5 days.

**Figure 4:**
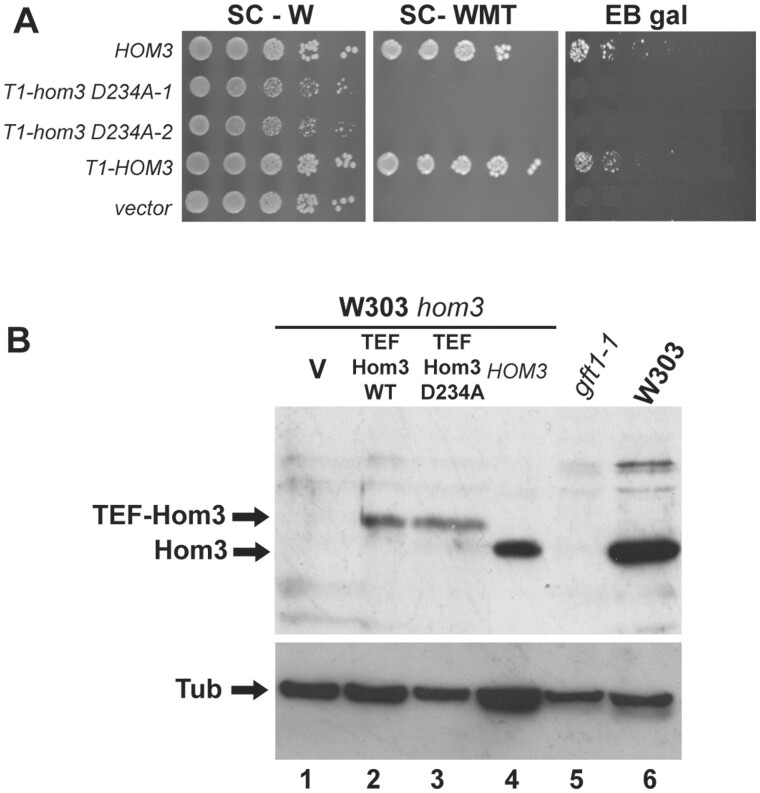
Aspartate kinase/Hom3 catalytic activity is required for LTA. (A) A *gal3 hom3* strain (ISY128) was transformed with plasmids bearing a genomic clone of *HOM3* (pIS297), a vector control (pRS314), T1-*HOM3* (pIS574) expressing the Hom3 ORF from the *TEF1* promoter, or two clones of pNH01 expressing the Hom3 D234A mutation. Cultures were diluted and spotted onto SC lacking tryptophan (SC-W), SC lacking tryptophan, methionine and threonine (SC-WMT), or EB gal, from 10-fold serial dilutions. (B) Protein extracts were prepared from WT W303-1A (lane 6), *gal3 gft1-1* (ISY135, lane5), or *hom3* (ISY279, lanes 1–4) strains transformed with plasmids expressing Hom3 from a genomic clone (pIS297, lane 4), Hom3 WT (pIS574, lane 2), the D234A mutant (pNH01, lane3) from the *TEF1* promoter, or a vector control (pRS314, lane 1). Samples were resolved on SDS-PAGE and analyzed by immunoblotting with antibodies against Hom3 (top panel) or tubulin (bottom). Arrows indicate migration of specific proteins produced from the *TEF1*-Hom3 and genomic Hom3 plasmids (top).

To identify regulators of Cdk8 activity, and phosphorylation of Gal4 at S699, we used a genetic screen to isolate mutants of genes necessary for growth of *gal3* yeast on EB gal. Because less than 10% of otherwise wild-type *gal3* yeast produce a colony on EB gal (Figure 2), we were unable to implement a conventional synthetic genetic array screen using the nonessential gene deletion collection for this purpose. Consequently, we employed UV-irradiation mutagenesis of a *gal3* W303 strain and replica plating, to identify mutants that were incapable of growth on EB gal, but which produced robust colonies on media containing three-carbon molecules as the sole carbon source. Initial mutants recovered from this process were transformed with a plasmid bearing genomic *GAL3* and re-assayed for growth on EB gal; mutants capable of growth when expressing Gal3 were designated the gal four throttle (*gft*) mutants, a collection which comprised several groups with distinct phenotypes that will be detailed in a separate report. The identity of one mutant, *gft1*, was determined by complementation with a plasmid library from WT yeast (Supplementary Figure S1), as a recessive allele of *hom3*, which encodes aspartate kinase. Transformation with plasmids bearing a genomic fragment of *HOM3*, or expressing the Hom3 ORF from the *TEF1* promoter, allow growth of the *gal3 gft1* strain on EB gal plates (Supplementary Figure S2). Additionally, disruption of *hom3* in a W303 *gal3* strain prevents growth on EB gal (Figure 3A), and also inhibits LTA to galactose as measured using a *GAL1*-GFP reporter (Figure 3B), or *GAL1*-LacZ reporter (Supplementary Figure S3). Furthermore, a *hom3* null allele was noncomplementing with the *gft1* mutation (data not shown), and we found that *gft1-1* mutant strains do not produce Hom3 protein as determined by immunoblotting (Figure 4B, lane 5). Consistent with these observations, sequencing of the *gft1-1* allele revealed a nonsense mutation at *HOM3* codon 473 (Supplementary Figure S4).

### LTA to galactose requires Hom3 catalytic activity but not homoserine biosynthesis

Aspartate kinase (Hom3) is an enzyme required for synthesis of homoserine, the precursor for threonine and methionine biosynthesis (Supplementary Figure S5). We determined whether a general defect in this pathway may prevent LTA of *gal3* yeast, by examining the effect of *hom2 and hom6* disruptions, which affect the enzymes aspartic beta semi-aldehyde dehydrogenase, and homoserine dehydrogenase, respectively, downstream of *HOM3* (Supplementary Figure S5). Neither of these gene disruptions affected growth on EB gal plates (Figure 3C), in either WT or *gal3* W303 yeast, indicating that a defect in *hom3* specifically impairs LTA in *gal3* yeast, rather than a general deficiency in this metabolic pathway. Additionally, we found that mutation of Hom3 aspartate 234 to alanine (D234A), a residue conserved within the catalytic domain of amino acid kinase enzymes (Marco-Marı’n *et al.* 2003), causes threonine and methionine auxotrophy, and also prevents growth of *gal3* yeast on EB gal plates (Figure 4A). The D234A *hom3* mutation does not affect abundance of Hom3 protein (Figure 4B), which indicates that Hom3/aspartate kinase catalytic activity is required for LTA and not merely the protein itself.

### Mutation of *hom3* causes a defect in TOR signaling

The Hom3 protein was previously shown to interact with the peptidyl-prolyl isomerase FKBP12/Fpr1, and this interaction is required for feedback inhibition of aspartate kinase activity by threonine (Alarcón and Heitman 1997). FKBP12 binds the immunosuppressive agent rapamycin, and this receptor ligand complex inhibits TORC1 kinase function by direct interaction (Heitman *et al.* 1991). We found that strains bearing the *gft1-1* mutation, and *hom3* disruption, were more sensitive to sub-lethal concentrations of rapamycin compared with wild-type W303 or a strain bearing a *gal3* disruption (Supplementary Figure S6A). This observation is consistent with previous analysis indicating that *hom3* mutants are amongst the most sensitive to rapamycin within the yeast haploid deletion set (Xie *et al.* 2005), and sensitivity to sub-lethal concentrations of rapamycin is known to indicate defects in TOR pathway signaling (Zurita-Martinez *et al.* 2007). Because of the previously described relationship between Hom3 and FKBP12 we examined whether this protein may also affect *GAL* expression, but found that disruption of *fpr1* did not affect growth of *gal3* W303 yeast on EB gal, nor did it suppress the effect of the *hom3* disruption on the *gft* phenotype (Supplementary Figure S6B).

**Figure 5:**
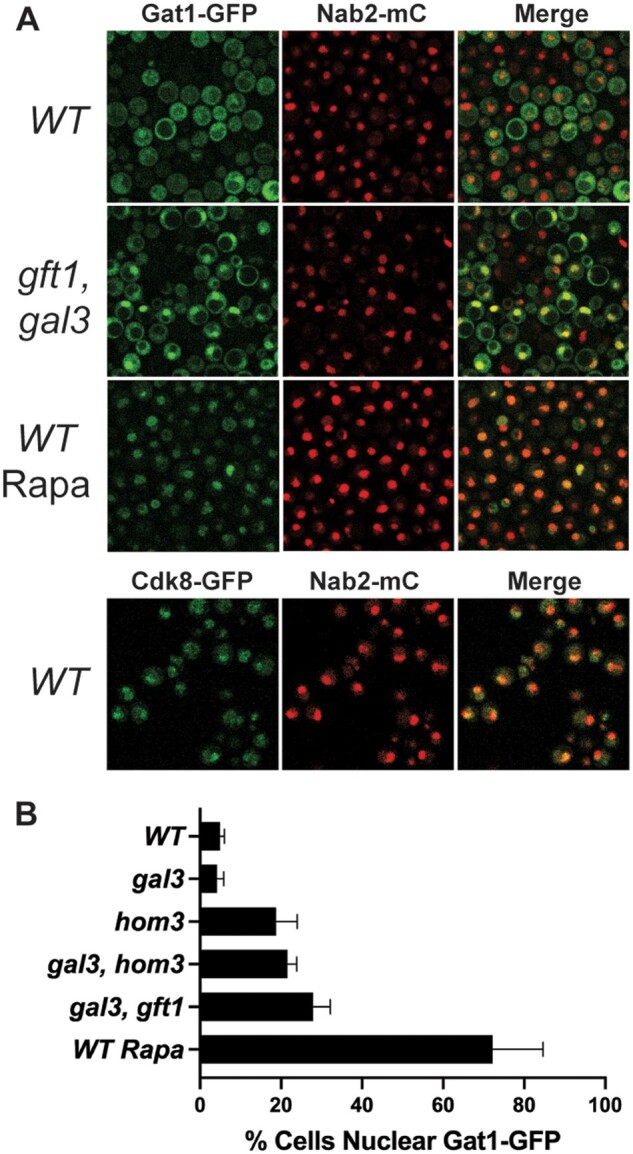
Defects in aspartate kinase/Hom3 promote nuclear localization of Gat1. (A) Wild-type W303 (WT) or *gft1 gal3* yeast expressing nuclear Nab2-mCherry and Gat1-GFP (top panels) or Cdk8-GFP (bottom panels) were grown in SC plus raffinose to OD *A*_600_ ∼ 0.8, and induced for 2 h with 2% galactose. Cells were harvested, washed with PBS, and analyzed by fluorescent confocal microscopy. Shown are the GFP (Gat1-GFP), mCherry (Nab2-mC) and merged images (Merge). WT cells (*WT* Rapa) were treated with 10 nM rapamycin during galactose induction. (B) Cells with the indicated genotype (or treatment, *WT* Rapa), expressing Nab2-mCherry and Gat1-GFP were prepared as in (A), and the % of cells displaying concentrated GFP that was co-localized with mCherry is indicated. Similar co-localization analysis was performed for Cdk8-GFP, were we observe 83% (±4) of WT cells express GFP co-localized with Nab2-mCherry.

Multiple nutrient responsive transcription factors, including the GATA factors Gat1 and Gln3 are negatively regulated by TOR signaling, where nutrient limitation, or treatment with rapamycin, causes their dephosphorylation allowing translocation to the nucleus for regulation of target genes (Georis *et al.* 2008). We examined the effect of a *hom3* null mutation, and the *gft1-1* allele, on subcellular localization of Gat1 using a GFP fusion (Figure 5). For this analysis we used strains that express Nab2-mCherry, which is constitutively localized to the nucleus (Zhu *et al*., 2019). Accordingly, we found that a Cdk8-GFP fusion protein predominately co-localized with Nab2-mCherry in wild-type W303 cells (Figure 5A, WT lower panels). Gat1-GFP expressed in untreated WT cells appears to be predominately cytoplasmic, and does not co-localize with Nab2-mCherry (Figure 5A, WT). However, greater than 70% of WT cells treated with rapamycin for 2 h displayed co-localized expression of Gat1 and Nab2 [Figure 5, A(WT Rapa) and B], consistent with previous observations indicating that inhibition of TOR signaling causes dephosphorylation of Gat1 to promote nuclear localization (Tate *et al.* 2015). Interestingly, we found that although less than 5% of WT W303 cells exhibit Gat1-Nab2 nuclear co-localization, cultures of strains bearing the *gft1-1* mutation, or disruption of *hom3* were found to produce co-localization of Gat1 and Nab2-mCherry in ∼20% of cells (Figure 5). In contrast, we did not observe this effect in a strain bearing a disruption of *gal3*, indicating that defects in Hom3/aspartate kinase promote nuclear localization of Gat1, which is indicative of inhibition of TOR signaling.

### Defects in TOR signaling prevent LTA to galactose

**Figure 6:**
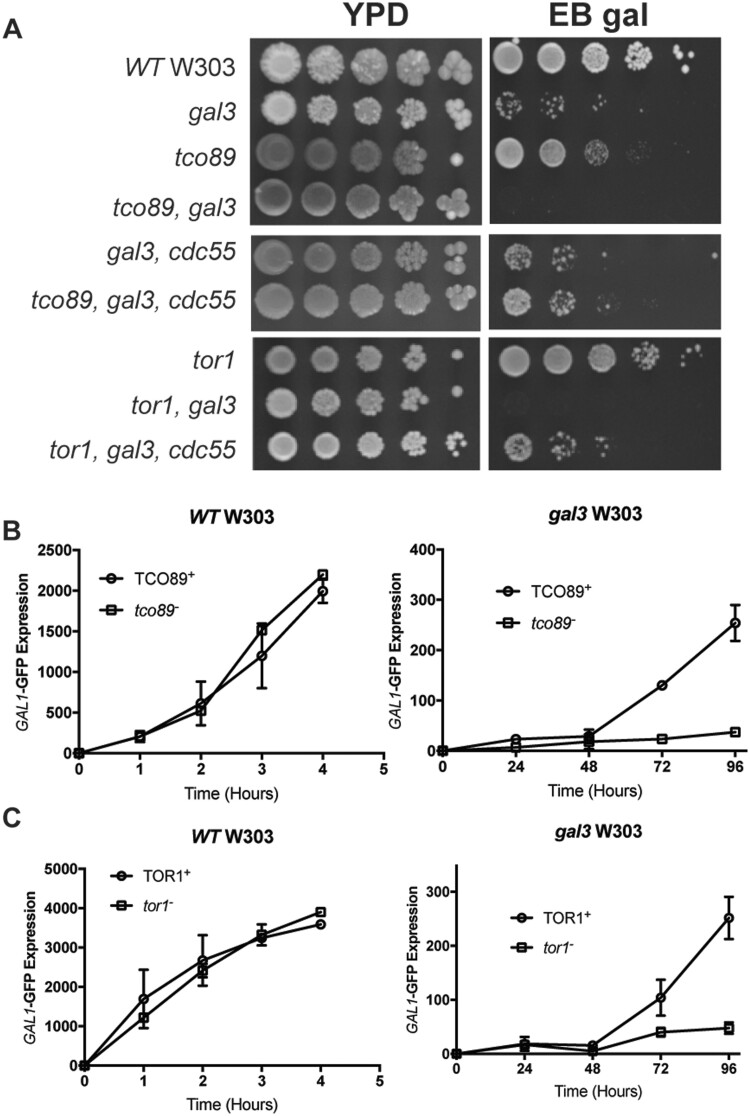
Mutation of TORC1 subunits prevents LTA in *gal3* yeast. (A) Strains with the indicated genotype were diluted, spotted onto YPD or EB gal plates in 10-fold serial dilutions, and grown at 30°C for 5 days. (B) WT (left panel) or *gal3* (right) yeast bearing WT *TCO89* (yNH029, yNH030) (○) or a *tco89* disruption (yNH035, yNH036) (□) were induced with 2% galactose for the indicated time (hours) and analyzed for GFP expression by flow cytometry. Results are presented as MFI of GFP expression and represent an average from three independent cultures. (C) WT (left panel) or *gal3* W303-1A (right) yeast bearing WT *TOR1* (yNH029, yNH030) (○) or a *tor1* disruption (yNH033, yNH034) (□) were induced with 2% galactose for the indicated time (hours) and analyzed by flow cytometry. Results are presented as MFI of GFP expression and represent an average from three independent cultures.

Because the *hom3* mutation caused hypersensitivity to sublethal concentrations of rapamycin, we subsequently found that five additional mutants from the *gft* collection produced this same phenotype, although amongst these *gft1*/*hom3* was the most sensitive (data not shown). Consequently, we examined whether disruption of genes encoding TOR signaling components also produce the *gft* phenotype in combination with a *gal3* mutation. The only nonessential genes encoding proteins of the TORC1 complex are *TOR1*, and *TCO89* (Loewith and Hall 2011). Interestingly, we found that disruption of *tco89* in a *gal3* W303 strain, prevents growth on EB gal plates, but disruption in a WT strain has no effect (Figure 6A). Consistent with the growth assays on EB gal, disruption of *tco89* on its own did not affect induction of a *GAL1*-GFP reporter gene (Figure 6B, left panel), but prevents induction in combination with a *gal3* disruption (Figure 6B, right panel), indicating that *tco89* disruption prevents LTA, equivalent to the effect of the *gft* mutants. Consequently, we examined whether any mutants from the *gft* screen might represent *tco89* alleles, where we found that *tco89* disruptions were noncomplementing with the mutant we had designated *gft7*; diploid cells produced by mating *gft7 and tco89* haploids did not produce spores capable of growth on EB gal, confirming that the *gft7* mutation is allelic to *tco89* (Supplementary Figure S7). Furthermore, sequencing of the *TCO89* ORF and 1 kb of flanking DNA revealed that the *gft7-1* mutant had a frameshift mutation that would be predicted to cause truncation of the protein at residue 639 (Supplementary Figure S8).

*TOR1* is nonessential for yeast growth, and is redundant with *TOR2* for TORC1 activity (Loewith and Hall 2011). We found that disruption of *tor1* in WT yeast did not affect growth on EB gal, but completely prevented growth of a *gal3* strain (Figure 6A), and thus a defect in *tor1* also produces a recessive *gft* phenotype (Supplementary Figure S9A), similar to the effect of *hom3 and tco89* mutants. Disruption of *tor1* also inhibits induction of *GAL*-GFP expression in a *gal3* strain (Figure 6C, right panel), but not in WT cells (left panel). However, unlike *tco89*, in complementation analysis we found that *tor1* disruption was not allelic to any of the mutants from our *gft* collection. Rather, we found that a *tor1* disruption was only partially complementing with the *gft2*, *gft6*, and *gft14* mutants, as these *tor1 gal3*/*gft gal3* heterozygous diploids typically did not grow on EB-gal as well as a *tor1 gal3*/*WT gal3* diploid (Supplementary Figure S9A). Furthermore, tetrad analysis of spores from these *tor gal3*/*gft gal3* heterozygotes produced *gft* mutant phenotype: *gal3* WT spores at a 3:1 ratio (Supplementary Figures S9B and S10), representing possible nonallelic noncomplementation. We confirmed this effect with analysis of a minimum of three tetrad sets produced from *tor1*/*gft2*, *gft6*, and *gft1*4 diploids (Supplementary Figure S10; data not shown). This indicates that these additional three *gft* mutants may also cause defects in TOR signaling. Importantly, we note that nonallelic noncomplementation was observed while characterizing the original *tor* mutants (Heitman *et al.* 1991). Taken together, these observations demonstrate that induction of the *GAL* genes by LTA in response to galactose requires TORC1, and specifically the Tor1 protein kinase.

### PP2A-Cdc55 phosphatase inhibits GAL induction

TOR signaling is known to inhibit several downstream protein phosphatases which control nutrient-responsive gene expression (Loewith and Hall 2011). Sit4 is a type 2A-related protein phosphatase regulated by TOR, and known to dephosphorylate multiple nutrient-responsive transcription factors, including Gat1 and Gln3 (Rohde *et al.* 2004; Georis *et al.* 2008). We examined whether Sit4 might modulate *GAL* induction but found that *sit4* disruption did not affect growth of wild-type or *gal3* yeast strains on EB gal (Supplementary Figure S11), indicating that Sit4 is not required for *GAL* induction. Furthermore, *sit4* disruption also did not allow growth of *hom3 gal3* yeast on EB gal, which suggests that Gal4 activity is likely not affected by Sit4 phosphatase (Supplementary Figure S11).

**Figure 7:**
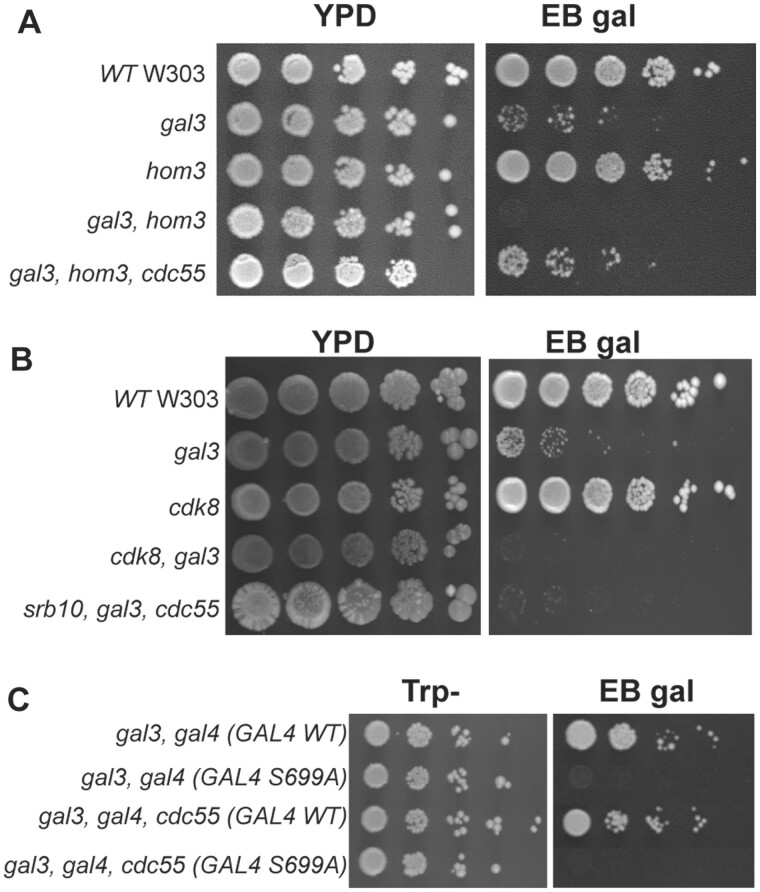
Disruption of *cdc55* suppresses effects of *hom3*, *tor1 and tco89* on LTA in *gal3* yeast. (A, B) Strains with the indicated genotype were grown overnight in YPD, diluted to an OD *A*_600_ of 1.0, spotted onto YPD or EB gal plates in 10-fold serial dilutions, and grown at 30°C for 5 days. (C) Strains *gal3 gal4* (YJR40) and *gal3 gal4 cdc55* (yNH037) were transformed with plasmids expressing WT GAL4 (YCpG4trp) or the GAL4 S699A mutant (pRD038), and cells spotted from liquid SC-Trp cultures in 10-fold serial dilutions on SC-Trp or EB gal plates, and grown for 5 days at 30°C.

TOR signaling also regulates protein phosphatase 2A (PP2A) comprised the redundant Pph21/Pph22 catalytic subunits and the nonessential Tpd3 and Cdc55 regulatory subunits (Jiang 2008). To examine if PP2A regulates *GAL* induction, we determined the effect of *cdc55* disruptions on growth of W303 strains on EB gal. Here we found that *cdc55* disruption had no effect on wild-type or *gal3* W303, but interestingly suppressed the *gft* phenotype of *gal3 hom3* mutants (Figure 7A). Similarly, we found that *cdc55* disruption also suppressed the effect of *tor1 and tco89* mutations for growth of *gal3* yeast on EB gal (Figure 6A), indicating that defects in TOR signaling for *GAL* expression must involve PP2A/Cdc55, which suggests that PP2A might counteract the effect of Cdk8 on Gal4.

Cdk8-dependent induction of *GAL* gene expression requires phosphorylation of Gal4 at serine 699 (Sadowski *et al.* 1996). Accordingly, disruption of *srb10*/*cdk8* has no effect on growth of WT yeast, but prevents growth of *gal3* yeast on EB gal (Figure 7B), which is typical of the *gft* phenotype described above. However, unlike *hom3*, *tor1*, and *tco89* mutants that affect TOR signaling, disruption of *cdc55* does not suppress the effect of *srb10*/*cdk8* mutants for growth of *gal3* yeast on EB gal (Figure 7B). Similarly, mutation of the Cdk8 phosphorylation site on Gal4 at S699 to alanine also prevents growth of *gal3* yeast on EB gal, and this effect is also not suppressed by disruption of *cdc55* (Figure 7C). These observations are consistent with the hypothesis that PP2A/Cdc55 must affect *GAL* induction by altering Cdk8-dependent phosphorylation of Gal4 at S699.

### Defects in Tor signaling inhibit Gal4 phosphorylation in vivo but not Cdk8 kinase activity

**Figure 8:**
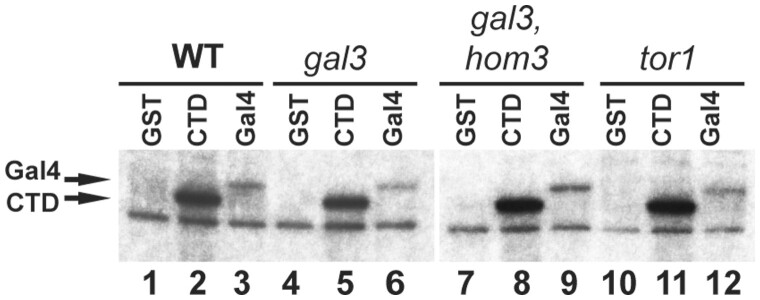
Mutations of *hom3 and tor1* did not affect Cdk8 kinase activity *in vitro*. Cdk8-FLAG was recovered from strains W303-1A, *gal3* (ISY54), *gal3 hom3* (ISY128), and *tor1* (yNH008) by immunoprecipitation and used for *in vitro* kinase assays with GST (lanes 1, 4, 7, 10), GST fused to RNAPII CTD (lanes 2, 5, 8, 11) or recombinant Gal4 protein (lanes 3, 6, 9, 12) as the substrate (Supplementary Figure S12). Reactions were analyzed by SDS-PAGE and autoradiography.

The *gft* genetic screen was devised to identify pathways required for Gal4 phosphorylation-dependent induction of *GAL* expression, and consequently, we expected that mutants would prevent phosphorylation at Gal4 S699 by causing inhibition of Cdk8 kinase activity, or enhancing dephosphorylation at this residue. To examine this, we transformed the mutant strains with a plasmid expressing Flag-epitope tagged Cdk8, and assayed kinase activity of complexes recovered by immunoprecipitation using GST, GST-RNA PolII C-terminal domain (CTD), and recombinant Gal4 protein as substrate. In these assays wild-type Cdk8-flag phosphorylates GST-CTD and Gal4, but not GST, but none of these substrates are phosphorylated by Cdk8 bearing a D290A mutation which inactivates kinase function (Hengartner *et al.* 1998) (Supplementary Figure S12). In these assays, we found that Flag-Cdk8 recovered from *gal3*, *hom3*, or *tor1* strains phosphorylated GST-CTD and Gal4 as efficiently as from wild-type cells (Figure 8), which indicates that inhibition of TOR signaling does not cause direct effects on Cdk8 kinase activity, at least as assayed *in vitro*. During characterization of the *gft* mutant collection, in contrast to results for *gft1*/*hom3*, we found that a separate class of mutants in the collection caused either partial or complete inhibition of Flag-Cdk8 activity in this assay (in progress).

**Figure 9:**
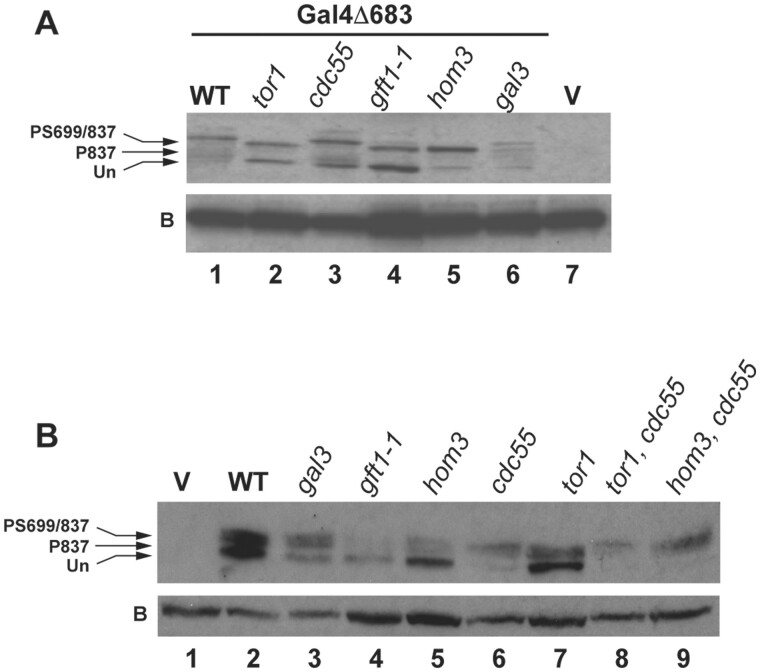
Mutation of *hom3 and tor1* inhibit Gal4 S699 phosphorylation *in vivo*, an effect that is reversed by disruption of *cdc55*. (A) Protein extracts from *gal80* W303-1A strains (WT, lanes 1 and 7, YKM001), *tor1* (YKM003), *cdc55* (YKM002), *gft1-1* (YKM004), *hom3* (YKM005), and *gal3* (ISY54) expressing the Gal4△683 derivative (YCpG4trp, lanes 1–6) or a vector control (pRS314, lane 7) were analyzed by immunoblotting with antibodies against Gal4 DBD. Arrows indicate migration of unphosphorylated Gal4△683 (Un), and species produced by phosphorylation at S837 and S699 (P699/837) or S837 (P837). Rabbit antibodies against Gal4 (DBD) produce a background signal (B) toward a yeast protein that is unaffected by any mutation or condition we have examined (Supplementary Figure S13). (B) Protein extracts from *gal80* strains with the indicated genotype expressing the Gal4△683 derivative (lanes 2–9) or a vector control (lane 1) were analyzed by immunoblotting with antibodies against Gal4 DBD.

We also examined the effect of *gft1*/*hom3 and tor1* mutations on Gal4 phosphorylation *in vivo*. Gal4 is phosphorylated on multiple sites which produce distinctive alterations in mobility in SDS-PAGE (Sadowski *et al.* 1996). This effect is particularly exaggerated with the Gal4△683 mutant bearing a large central deletion, retaining only the N-terminal DNA-binding domain (1–147) and C-terminal region including all of the verified *in vivo* sites of phosphorylation (683–881) (Sadowski *et al.* 1996). Additionally, because Gal4’s phosphorylation occurs consequential to transcriptional activation (Sadowski *et al.* 1991) we expressed this protein in a *gal80* background where Gal4 activates transcription constitutively. The Gal4△683 protein produces multiple species detected by immunoblotting, phosphorylation of the regulatory S699 produces the slowest migrating species, in combination with S837 phosphorylation; accordingly, this species is absent in cells bearing mutation of *cdk8* (Hirst *et al.* 1999) (Supplementary Figure S13). We observe the slowest migrating species in WT yeast (Figure 9A, lane 1) as well as strains bearing disruption of *gal3* (lane 6) or *cdc55* (lane 3). However, we note that strains bearing disruption of *tor1* (lane 2), *hom3* (lane 5), or the *gft1-1* mutation (lane 4) produce a faster migrating species typical of loss of the Cdk8-dependent P-S699 phosphorylation (Sadowski *et al.* 1996) and loss of Cdk8 function (Supplementary Figure S13). These observations indicate that defects in Tor signaling inhibit the appearance of phosphorylation of Gal4 at S699 *in vivo*. Furthermore, in *tor1 and hom3* strains that also bear disruption of *cdc55*, we observe only the slowest migrating form, indicating that loss of Cdc55/PP2A suppresses the effect of TORC1 signaling defects on Gal4 phosphorylation, and support the contention that phosphorylation of Gal4 at S699 might be dephosphorylated by Cdc55/PP2A.

### Inhibition of aspartate aminotransferase (Aat1/2) suppresses the effect of *hom3* mutation on GAL induction

**Figure 10:**
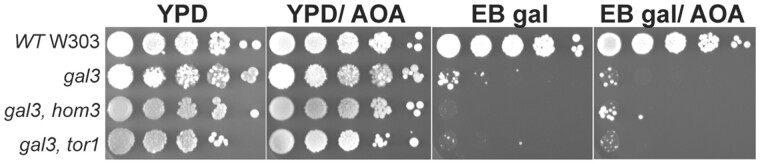
Inhibition of aspartate aminotransferase (Aat1/2) allows growth of *gal3 hom3* yeast on EB gal. Yeast strains with the indicated genotype were grown overnight in YPD, diluted to an OD *A*_600_ of 1.0, and spotted in 10-fold serial dilutions on YPD or EB gal plates, or YPD and EB gal containing 10 nM aminooxyacetic acid (YPD/AOA, EB gal/AOA).

Mutations of *hom3* cause sensitivity to sub-lethal concentrations of rapamycin (Supplementary Figure S6A) (Xie *et al.* 2005), and defects in TORC1 signaling (Figure 5) which raises the question as to how defects in aspartate kinase produce this effect on TOR. One possibility is that defective Hom3 function may cause accumulation of intracellular levels of aspartate (Supplementary Figure S5), which might cause inhibition of TOR signaling. To examine this possibility, we determined whether elevated concentrations of aspartate affected growth of *gal3* yeast on EB gal, but did not observe an effect (data not shown). However, we note that addition of excess aspartate to the growth medium is unlikely to affect intracellular concentrations without additional overexpression of the corresponding transporter (Bianchi *et al*., 2019; Ruiz *et al*., 2020). Aspartate aminotransferase, encoded by the redundant genes *AAT1*/*AAT2*, catalyzes synthesis of aspartate from oxaloacetate, and this reaction is inhibited by AOA (Supplementary Figure S5) (Antti and Sellstedt, 2018). Interestingly, we found that AOA had no effect on growth of yeast on YPD (Figure 10, YPD/AOA), but suppressed the effect of the *hom3* mutation for growth of *gal3* yeast on EB gal (EB Gal/AOA). In contrast, AOA had no effect on growth of *tor1 gal3* yeast on EB gal. This observation indicates that inhibition of aspartate biosynthesis reverses the effect of *hom3* mutation on growth of *gal3* yeast, suggesting that accumulation of aspartate may inhibit TOR signaling.

## Discussion

**Figure 11:**
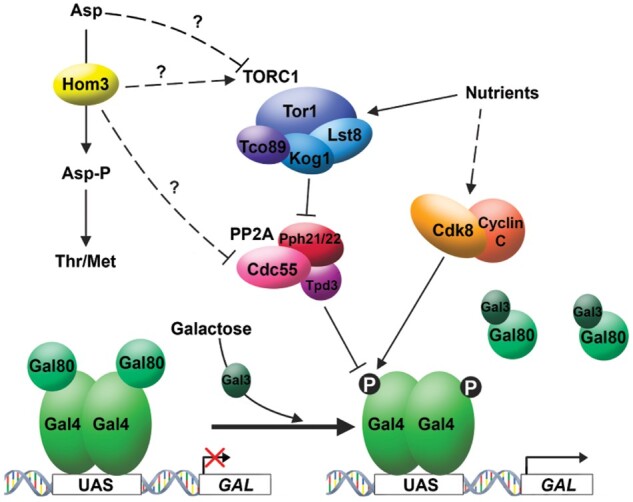
*GAL* induction is modulated by TOR signaling through PP2A/Cdc55. Galactose induces *GAL* gene expression by binding the inducer protein Gal3, which relieves the inhibitory effect of Gal80 on the transactivator Gal4. Gal4 is phosphorylated on S699 by Cdk8 during transactivation, which is required for full *GAL* gene induction. Nutrients modulate *GAL* induction through TORC1 signaling, and PP2A/Cdc55 likely inhibits Gal4 activity by dephosphorylation of the Cdk8-dependent site at S699. Previous observations, and phenotypes produced by additional classes of mutants from the *gft* genetic screen indicate that Cdk8 activity itself is also regulated by nutrient signaling.

The yeast *GAL* genes represent an important model for understanding eukaryotic gene regulation in response to signal transduction. In this study, we exploited the LTA/delayed *GAL* gene induction phenotype of *gal3* yeast, which is Cdk8-dependent (Rohde *et al.* 2000), to identify regulators of Cdk8-dependent phosphorylation of Gal4 using a mutant screen. This screen identified multiple mutants that affect TORC1 signaling, which indicates that expression of the *GAL* genes is modulated by nutrient signals that control TORC1 activity. Our results indicate that defects in TORC1 signaling do not directly affect Cdk8 kinase activity, but rather cause enhanced dephosphorylation of the Cdk-dependent phosphorylation site on Gal4 at S699 through hyperactivation of PP2A/Cdc55. These observations are consistent with known effects caused by inhibition of TORC1 activity, typified by dephosphorylation of transcription factors involved in nutrient response, including Gln3 and Gat1 which enables their nuclear translocation and activation of genes required for nutrient response (Georis *et al.* 2008). Comparably, full induction of the *GAL* genes is dependent upon phosphorylation of Gal4 at S699 (Hirst *et al.* 1999), and consequently given our results, it seems likely that PP2A hyperactivation where TOR signaling is impaired inhibits Cdk8-dependent *GAL* induction through dephosphorylation of Gal4 P-S699, which would implicate this specific phosphorylation as a regulatory substrate for PP2A (Figure 11). However, unlike Gat1 and Gln3, whose nuclear localization and activity are largely dependent upon the TORC1-regulated phosphatases PP2A and Sit4 (Tate *et al.* 2015), activity of Gal4 is likely to be fine-tuned by PP2A-dependent dephosphorylation to adjust *GAL* expression to levels appropriate for the growth environment.

Regulation of TOR signaling is complex and involves sensing of nutrient availability through intracellular abundance of amino acids, most particularly glutamine (Tate *et al.* 2015). A relationship between aspartate kinase and TOR signaling was noted previously, in that *hom3* disruptions were found amongst the most sensitive to sub-lethal concentrations of rapamycin within the nonessential yeast deletion set (Xie *et al.* 2005). Furthermore, Hom3 physically interacts with the rapamycin receptor FKBP12/Fpr1, which was shown to modulate feedback inhibition of Hom3 activity by threonine (Alarcón and Heitman 1997), and therefore it is possible that Hom3 protein may have a regulatory function for TORC1 (Figure 11). Impairing Hom3 catalytic activity by mutation produces the *gft* phenotype, as does *hom3* disruption, but this effect is not produced by mutation of downstream genes involved in homoserine biosynthesis (Supplementary Figure S5). These observations indicate that specific loss of Hom3 catalytic activity, rather than general loss of this metabolic pathway causes inhibition of TOR signaling, although interestingly hyper-sensitivity to rapamycin appears to be typical of mutations of all gene components of this pathway (Xie *et al.* 2005). However, the fact that the *gft* phenotype is only produced by *hom3* mutation suggests that aspartate kinase has an additional, more elaborate regulatory role for TOR signaling. Interestingly, a previous relationship between homoserine biosynthesis and Cdk8 activity was noted in that accumulation of β-aspartate semialdehyde caused by disruption of *hom6* (Supplementary Figure S5) promotes both Cdk8 and Pho85-dependent degradation of Gcn4, but the mechanism for this effect has not been established (Rawal *et al.* 2014). Our results indicate that accumulation of aspartate in yeast defective for *hom3* might cause inhibition of TOR signaling (Figures 10 and 11). This is consistent with an observation that aspartate inhibits kinase activity in a permeabilized cell assay for TORC1 (Tanigawa and Maeda 2017), and that inhibition of aspartate synthesis from oxaloacetate (Supplementary Figure S5) suppresses the effect of a *hom3* deletion on *GAL* expression (Figure 10). Hom3 protein was also identified in complexes with Cdc55 in affinity capture MS proteomics studies (Breitkreutz *et al.* 2010); the significance of this interaction is unknown, but it is possible that Hom3 may also regulate Cdc55/PP2A by direct interaction (Figure 11). Overall, our results illustrate an importance of Hom3/aspartate kinase for TORC1 signaling, but the molecular details of this regulatory relationship have yet to be elucidated.

The function of Cdk8 and the partially redundant Cdk19 protein kinase in humans are of considerable interest because a significant proportion of cancers may have alterations in their activity, particularly of melanoma and colorectal origin (Nemet *et al.* 2014). Cdk8/19 have enigmatic biological effects because phosphorylation of their target substrates have positive or negative effects on activity of specific factors and transcription of target genes (Firestein and Hahn 2009). Currently, there are no known upstream regulators of Cdk8 kinase activity. The *gft* mutant screen was designed to address this issue, and characterization of one mutant revealed a role of TOR signaling for regulation of *GAL* gene expression. The LTA phenotype is among the earliest phenotypic differences described between various laboratory strains of *Saccharomyces cerevisiae*, and extensive analysis of this phenotype had indicated an important role for nutrient signaling regulating delayed *GAL* gene induction (Rohde *et al.* 2000). Consequently, our discovery that TOR signaling modulates induction of *GAL* expression is consistent with these previous observations. Considering that these signaling molecules are conserved amongst eukaryotes, it will be interesting to determine whether mTORC1 activity may regulate Cdk8/19 target factors in human cells. Characterization of additional mutants of the *gft* collection indicate that nutrient signaling must also regulate Cdk8 activity directly (Figure 11), and consequently the identity of these will provide significant further insight into regulation of Cdk8 function in eukaryotes.

## Data availability

All data relating to this article are available in the article and in the Supplementary Material. All strains and plasmids described in the article are available upon request.

Supplementary material is available at *GENETICS* online.

## Supplementary Material

iyab168_Supplementary_Data
