# Supplementary material for: TORC1 signaling modulates Cdk8-dependent GAL gene expression in Saccharomyces cerevisiae
Source: Genetics. 2021 Oct 5;219(4):iyab168. doi: 10.1093/genetics/iyab168 (PMC8664586; doi:10.1093/genetics/iyab168)
Supplement: iyab168_Supplementary_Data [file iyab168_supplementary_data.pdf]

Legends to Supplementary Figures

Figure S1: Clones of WT genomic DNA from chromosome V spanning the *HOM3* region compliment the *gft1-1* mutation. Plasmids from a genomic library in pRS314 prepared from WT yeast were recovered from transformants of a *gft1-1* strain that were capable of growth on EB gal, as described in Materials and Methods. Shown is a graphical representation of genes on chromosome V spanning the region between *GIP2* and *TPA1*. Green bars (below) indicate genomic DNA sequences spanning the insert of recovered clones as determined by sequencing. One isolate each of clone 1 (pIS297) and clone 2 (pIS298), were recovered, and 2 isolates of clone 3 (pIS299) in the complementation analysis.

Figure S2: Plasmids expressing *HOM3* complement the growth defect of *gft1-1 gal3* yeast on EB gal. Yeast strain ISY135, bearing the *gft1-1* mutation was transformed with plasmids, including a vector control (*vector*, pRS314), expressing Hom3-3XFlag expressed from the *TEF1* promoter (pIS556, T1-*HOM3-Flag*), bearing a genomic clone of *HOM3*, recovered in complementation analysis of the mutant phenotype (pIS297, *pHOM3*), or vectors expressing the WT (pIS574, *T1-HOM3*) or D234A mutant (pNH01, *T1-hom3* D234A) ORF from the *TEF1* promoter. Cultures were diluted and spotted onto SC lacking tryptophan (SC-W), SC lacking tryptophan, methionine and threonine (SC-WMT), or EB gal, from 10-fold serial dilutions.

Figure S3: Mutation of *hom3* inhibits induction of *GAL1* expression in *gal3* yeast. W303-1A (WT, left) and (*gal3*, right) yeast expressing WT HOM3 (●) or a *hom3* deletion (□) and

bearing a *GAL1*-LacZ reporter were induced with 2% galactose for the indicated times (hrs), and assayed for β-galactosidase activity. Results represent an average of 3 determinations.

Figure S4: Sequence analysis of the *gft1-1* mutation. Genomic DNA prepared from yeast bearing the *gft1-1* mutation was used as template for PCR amplification with oligos IS2897/ IS2898; the resulting product was digested XhoI/ NotI and cloned into pGEM11. Three independent clones were analysed by sequencing using a nested set of forward and reverse primers specific for *HOM3*. Shown are Sanger sequencing chromatograms spanning a region bearing the only sequence difference identified between *gft1-1* and WT DNA.

Figure S5: Biosynthetic pathway in yeast for homoserine, threonine and methionine. The metabolic pathway for biosynthesis of threonine and methionine from pyruvate is illustrated, and genes encoding enzymes for production of homoserine are indicated, including *PYC1/2* (pyruvate carboxylase), *AAT1/2* (aspartate aminotransferase), *HOM3* (aspartate kinase), *HOM2* (aspartic beta semi-aldehyde dehydrogenase) and *HOM6* (homoserine dehydrogenase). Aspartate aminotransferase activity is inhibited by aminooxyacetic acid (AOA).

Figure S6: Disruption of *hom3* causes sensitivity to sub-lethal concentrations of rapamycin. Panel A: Strains with the indicated genotype were spotted from overnight YPD cultures in 10-fold serial dilutions YPD or YPD containing 5 ng/mL rapamycin plates and incubated at 30°C for 3 days. **Panel B:** Strains with the indicated genotype were grown overnight in YPD, diluted to an O.D. A_{600} of 1.0, spotted onto YPD or EB gal plates in 10-fold serial dilutions, and grown at 30°C for 5 days.

Figure S7: The *gft7-1* mutation is allelic to *tco89*. Diploid strains were produced by mating *gal3 tco89* (yNH017) with *gal3* (ISY54) (top panel), or *gal3 gft7-1* (ISY192) (bottom panel). Following sporulation, tetrads were dissected and haploids from spores were spotted from YPD cultures at 10-fold serial dilutions onto YPD or EB gal plates. The plates were incubated for 5 days at 30°C.

Figure S8: Sequence analysis of the *gft7-1* mutation. Genomic DNA prepared from a *gft7-1* strain was used for PCR amplification with oligos IS2899 and IS2900. The resulting product was digested with BamHI/ NotI and cloned into pGEM11. Three independent clones were analysed by sequencing using a nested set of forward and reverse primers specific for *TCO89*. Shown are Sanger sequencing chromatograms spanning a region bearing the only sequence difference identified between *gft7-1* and WT DNA.

Figure S9: Disruption of *tor1* produces unlinked non-complementation with *gft2-1* and *gft14-1*. Panel A: W303 derived yeast strains were grown overnight in YPD, diluted to an O.D. A_{600} of 1.0, and spotted onto YPD or EB gal plates from 10 fold serial dilutions. Haploid stains include *WT*, *gal3*, and *gal3 hom3* W303. Diploid strains bearing disruption of *tor1* and *gal3* were produced from crosses with *MAT*α *gal3* WT (*tor1, gal3*/ *gal3*), or strains bearing the *gft2* (*tor1, gal3*/ *gft2*, *gal3*), *gft6* (*tor1, gal3*/ *gft6*, *gal3*), and *gft14* (*tor1, gal3*/ *gft14*, *gal3*), mutant strains. **Panel B:** Diploids were produced by mating of *MAT***a** *gal3 tor1* yeast with *MAT*α *gal3* WT (*tor1*, *gal3*/ *gal3*), *gft2-1* (*tor1*, *gal3*/ *gal3, gft2*), or *gft14-1* (*tor1*, *gal3*/ *gal3, gft14*) haploid strains. Tetrads were dissected from sporulated cultures of the resulting diploids, and haploids

from spores spotted onto YPD or EB gal plates. The plates were incubated for 5 days at 30°C. Results shown are representative of 3 tetrad dissections.

Figure S10: Disruption of *tor1* produces unlinked non-complementation with *gft6-1*. Diploid strain YKM017, produced by mating of *MAT***a** *gal3 tor1* yeast with *MAT*α *gal3 gft6-1*. Four sets of tetrads were dissected from sporulated and haploids from spores spotted onto YPD, EB gal, SC-his (H), and YPD+G418 (K) plates, and inducted for 5 days at 30°C. Shown are results from tetrad sets 3 and 4.

Figure S11: *GAL* expression in *gal3* yeast is unaffected by disruption of *sit4*. Strains with the indicated genotype were grown overnight in YPD, diluted to an O.D. A_{600} of 1.0, spotted onto YPD or EB gal plates in 10-fold serial dilutions, and grown at 30°C for 5 days.

Figure S12: Assay of Cdk8 kinase activity *in vitro*. Cdk8-FLAG WT (Cdk8 WT, lanes 1-6) or kinase inactive Cdk8 D290A-FLAG (lanes 7-9) was recovered from yeast by immunoprecipitation with anti-FLAG antibody, and used for *in vitro* kinase assays with GST (lanes 1, 4, 7), GST fused to RNAPII CTD (lanes 2, 5, 8) or recombinant Gal4 protein (lanes 3, 6, 9) as substrate and 2 pmol [γ-32P]ATP. Reactions were resolved by 10% SDS-PAGE, and the gels stained with coomassie blue (lanes 1-3). Gels were dried and exposed to Kodak Biomax film (lanes 3-9). Migration of Gal4, GST-CTD (CTD) and GST protein substrates, and antibody heavy (H) and light (L) chains are indicated (left).

Figure S13: Phosphorylation of the Gal4Δ683 deletion derivative. Protein extracts from *gal80* W303-1A (lanes 1-4) or cdk8 gal80 (lanes 5-6) strains were analyzed by immunoblotting with antibodies against Gal4 DBD. Cells expressed wild type Gal4Δ683 (lanes 2 and 6), Gal4Δ683 S699A (lane 3), Gal4Δ683 S837A (lanes 4 and 5), or a vector control (lane 1). Note that Gal4 also becomes phosphorylated at S691 and S696, modifications which are detected with the Gal4Δ683 S699A derivative (Sadowski *et al.* 1996).

Figure S1

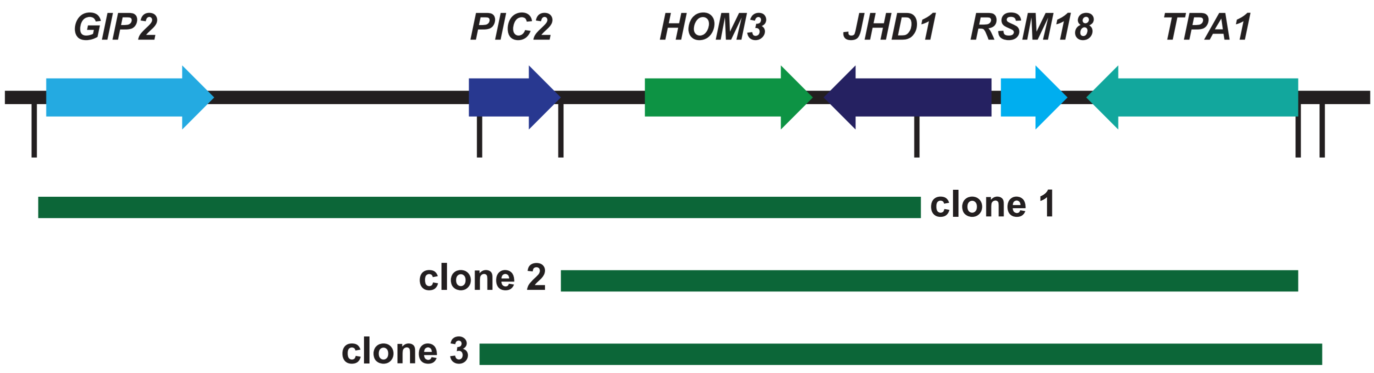

Figure S2

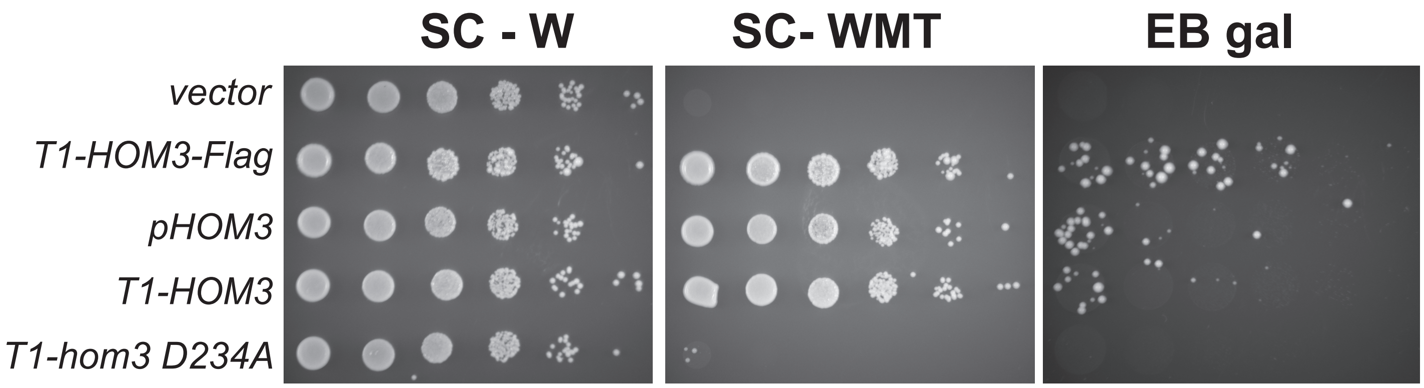

SC - W
SC- WMT
EB gal
vector
T1-HOM3-Flag
pHOM3
T1-HOM3
T1-hom3 D234A

Figure S3

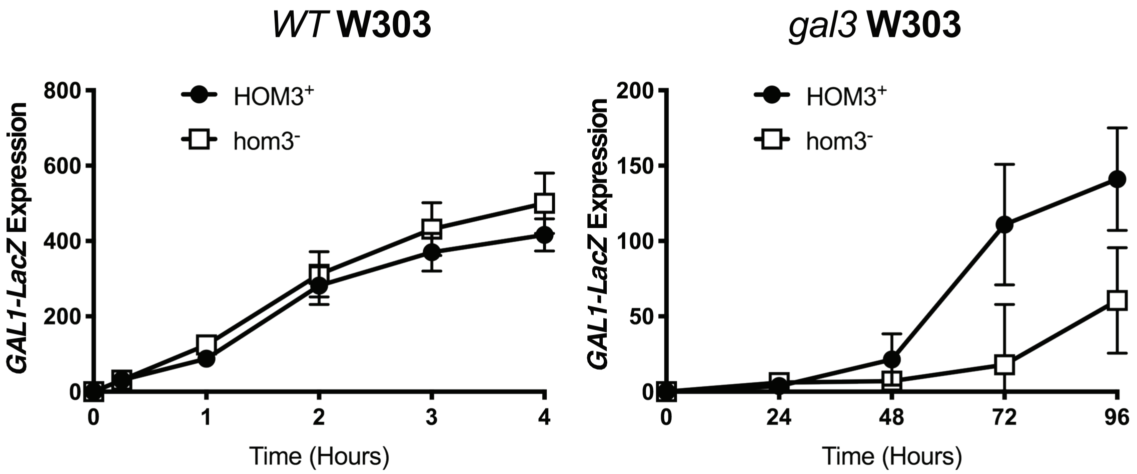

WT W303
gal3 W303
GAL1-LacZ Expression
HOM3+
hom3-
800
600
400
200
0
0
1
2
3
4
Time (Hours)
200
150
100
50
0
0
24
48
72
96
Time (Hours)

Figure S4

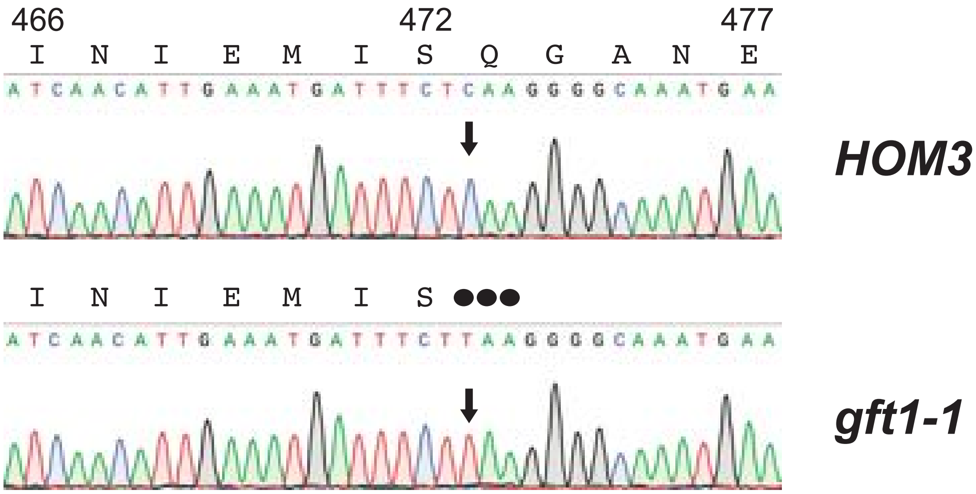

466
472
477
I N I E M I S Q G A N E
ATCAACATTGAAATGATTTCTCAAGGGGCAAATGAA
HOM3
I N I E M I S ●●●
ATCAACATTGAAATGATTTCTTAAGGGGCAAATGAA
gft1-1

Figure S5

Biosynthesis of homoserine, threonine and methionine

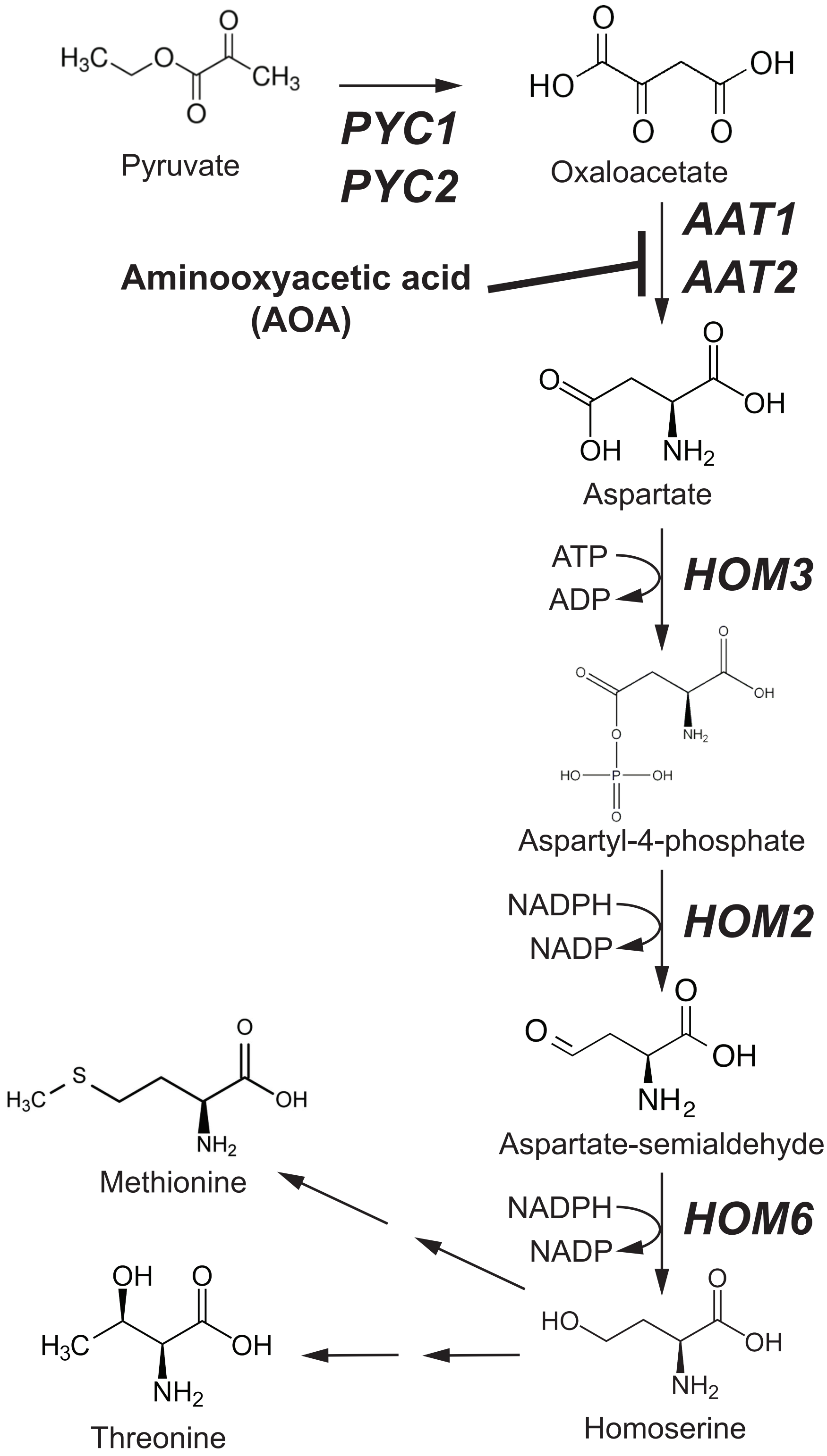

Figure S6

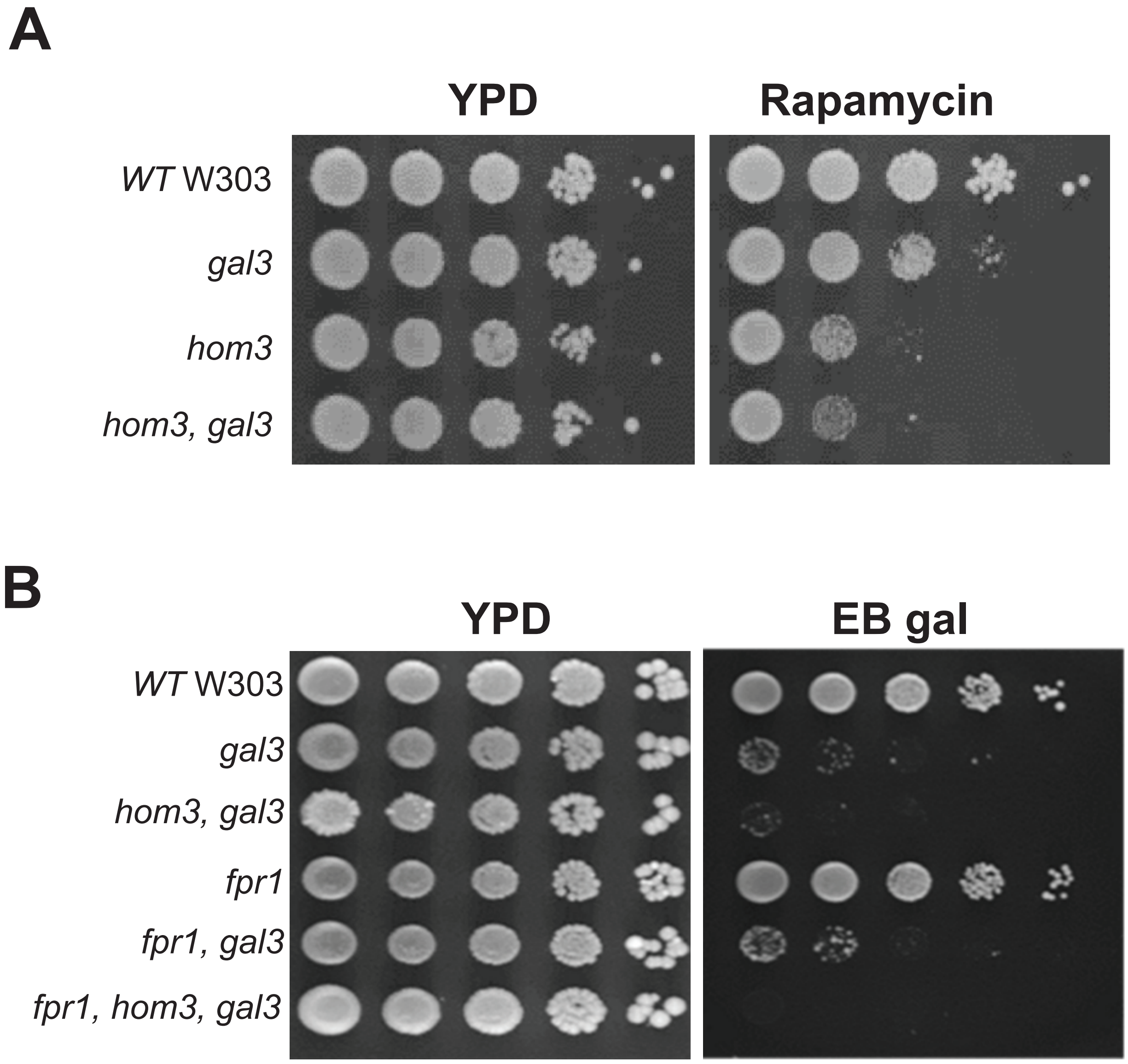
A
YPD
Rapamycin
WT W303
gal3
hom3
hom3, gal3
B
YPD
EB gal
WT W303
gal3
hom3, gal3
fpr1
fpr1, gal3
fpr1, hom3, gal3

Figure S7

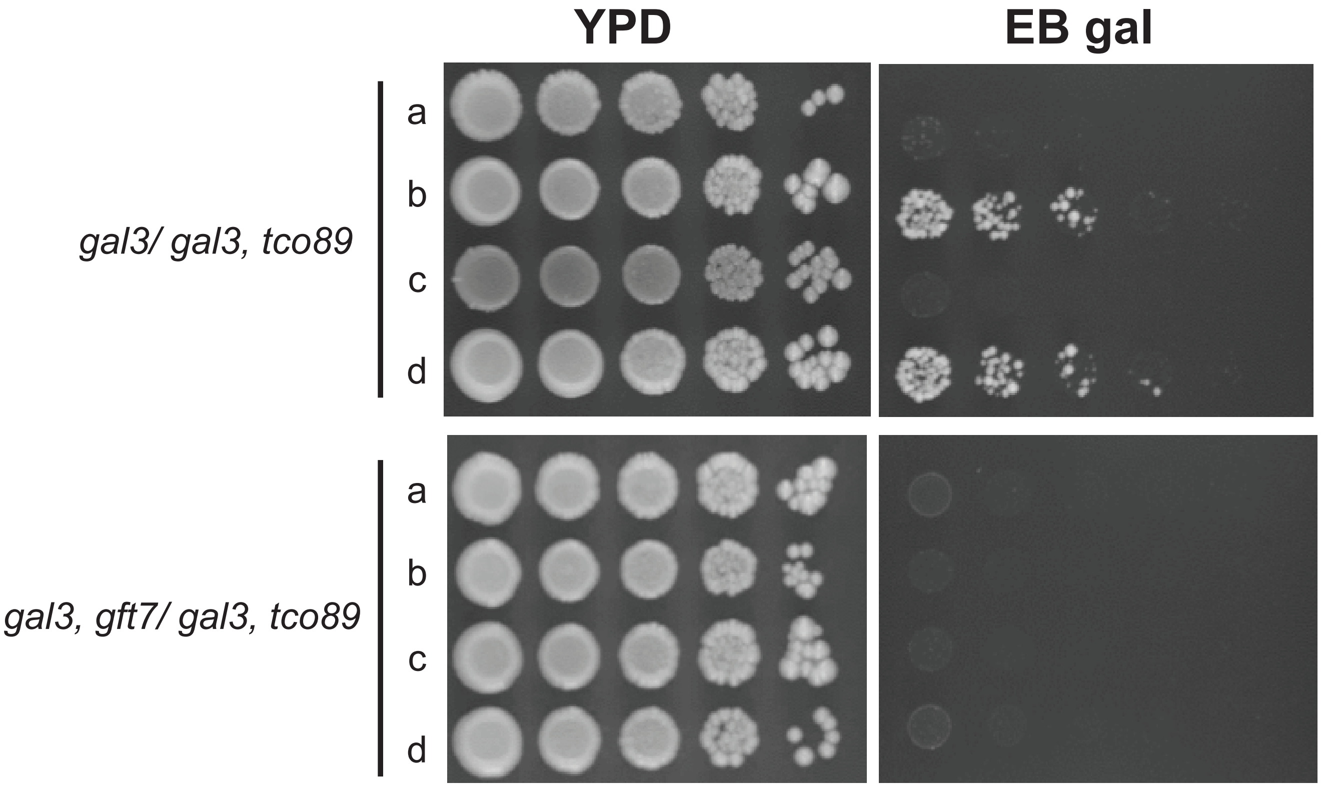

Figure S8

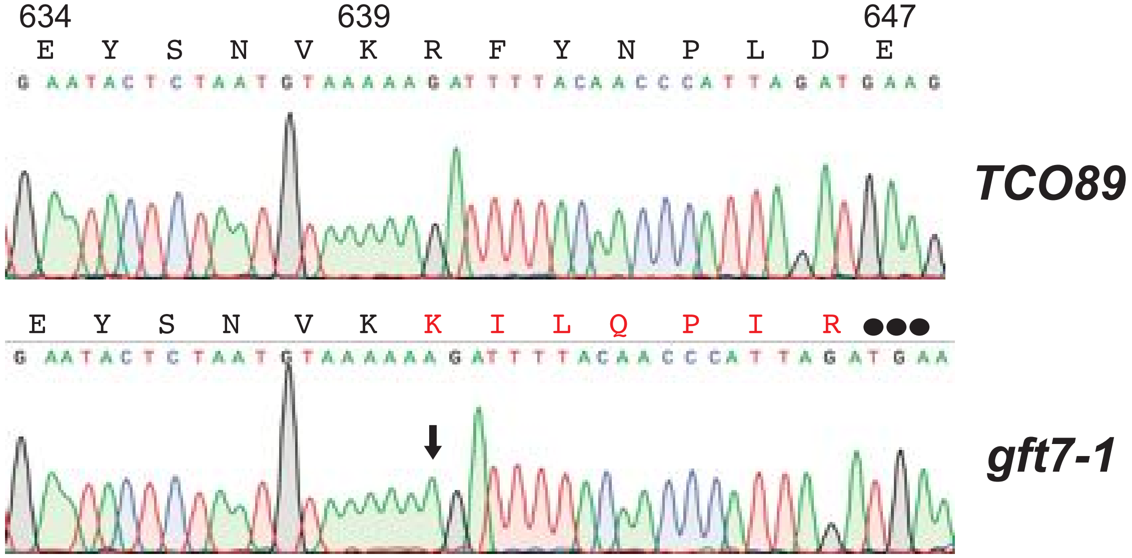

634
639
647
E Y S N V K R F Y N P L D E
G AATACTCTAATGTAAAAAGATTTTACAACCCATTAGATGAAG
TCO89
E Y S N V K K I L Q P I R
G AATACTCTAATGTAAAAAAGATTTTACAACCCATTAGATGAA
gft7-1

Figure S9

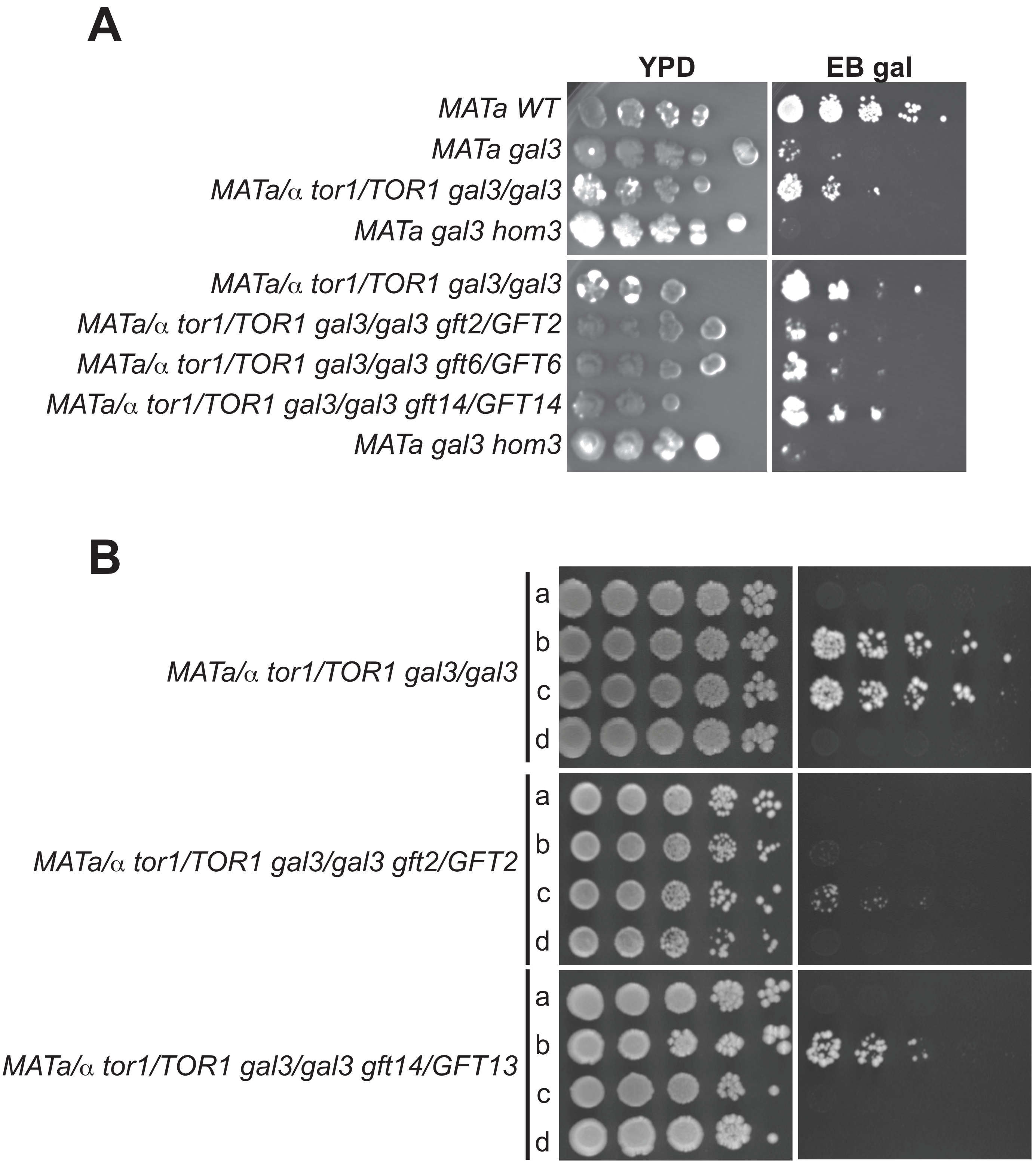
A
YPD
EB gal
MATa WT
MATa gal3
MATa/α tor1/TOR1 gal3/gal3
MATa gal3 hom3
MATa/α tor1/TOR1 gal3/gal3
MATa/α tor1/TOR1 gal3/gal3 gft2/GFT2
MATa/α tor1/TOR1 gal3/gal3 gft6/GFT6
MATa/α tor1/TOR1 gal3/gal3 gft14/GFT14
MATa gal3 hom3
B
a
b
c
d
MATa/α tor1/TOR1 gal3/gal3
a
b
c
d
MATa/α tor1/TOR1 gal3/gal3 gft2/GFT2
a
b
c
d
MATa/α tor1/TOR1 gal3/gal3 gft14/GFT13

Figure S10

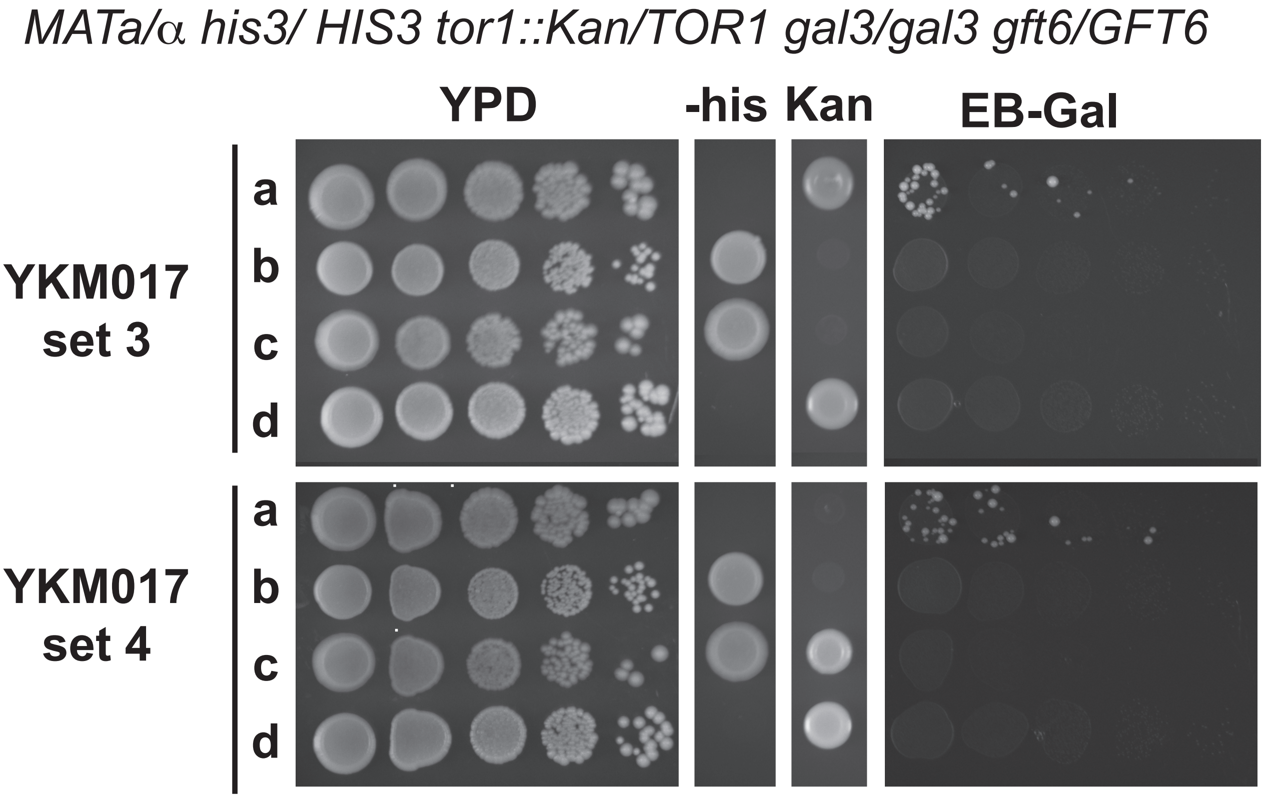

MATa/α his3/ HIS3 tor1::Kan/TOR1 gal3/gal3 gft6/GFT6
YPD
-his
Kan
EB-Gal
YKM017 set 3
a
b
c
d
YKM017 set 4
a
b
c
d

Figure S11

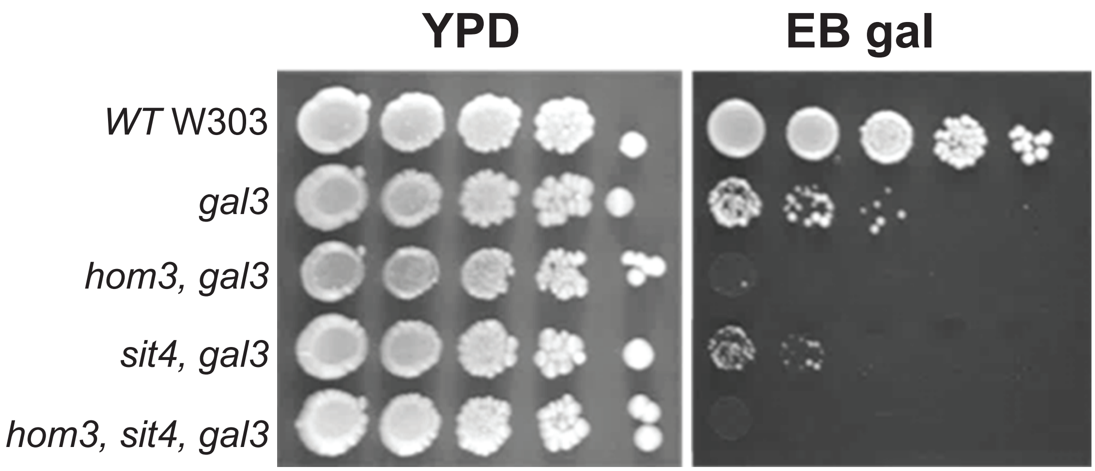

Figure S12

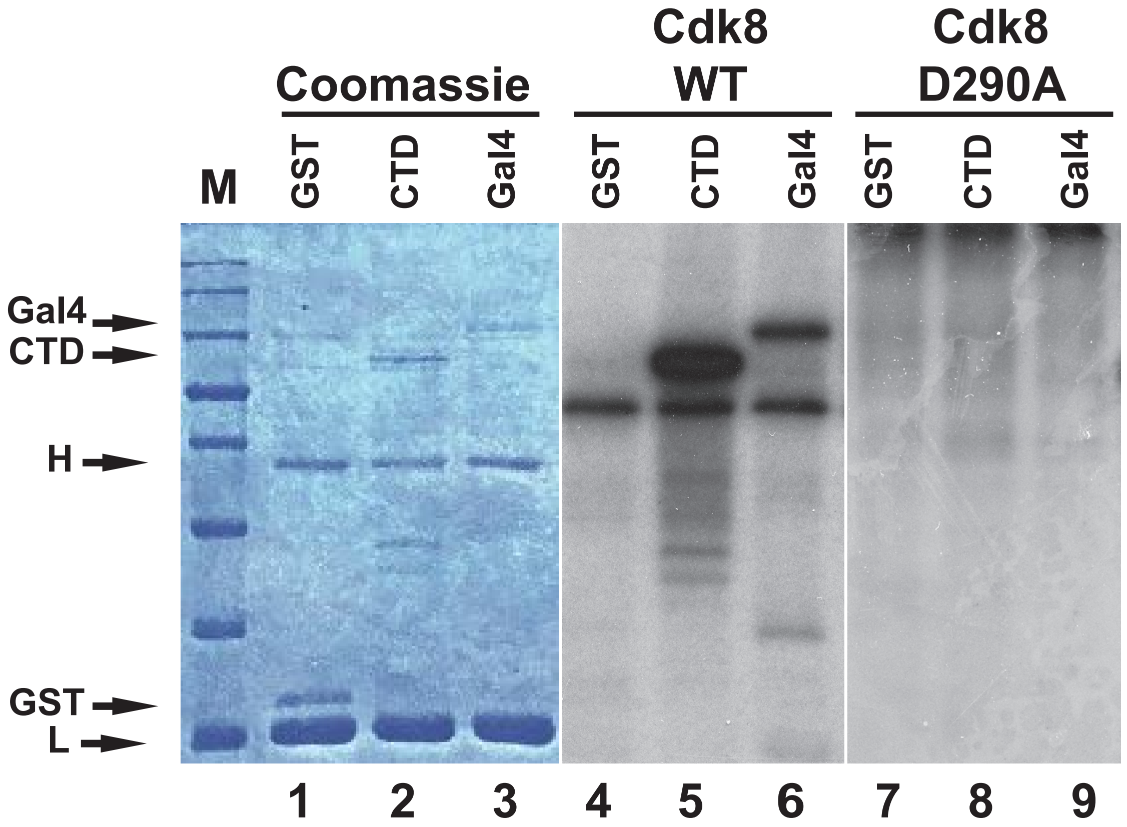

Coomassie
Cdk8
WT
Cdk8
D290A
M
GST
CTD
Gal4
GST
CTD
Gal4
GST
CTD
Gal4
Gal4
CTD
H
GST
L
1
2
3
4
5
6
7
8
9

Figure S13

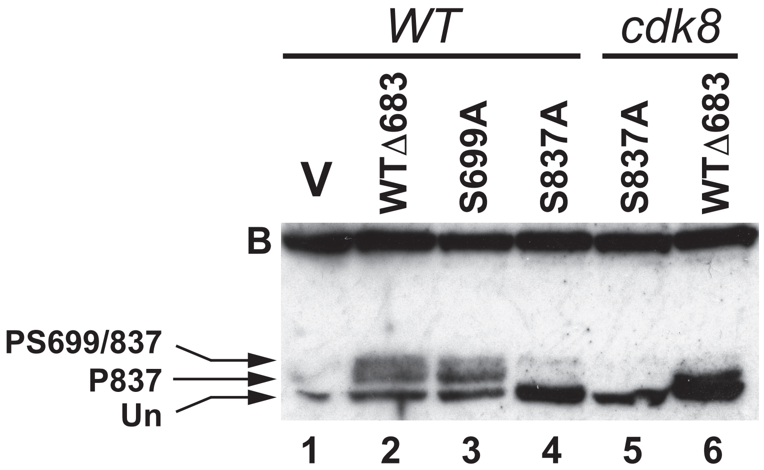

WT
cdk8
V
WTΔ683
S699A
S837A
S837A
WTΔ683
B
PS699/837
P837
Un
1
2
3
4
5
6
